# Machine learning-enhanced drug testing for simultaneous morphine and methadone detection in urinary biofluids

**DOI:** 10.1038/s41598-024-58843-9

**Published:** 2024-04-06

**Authors:** Mohammad Mehdi Habibi, Mitra Mousavi, Maryam Shekofteh-Gohari, Anita Parsaei-Khomami, Monireh-Alsadat Hosseini, Elnaz Haghani, Razieh Salahandish, Jahan B. Ghasemi

**Affiliations:** 1 School of Chemistry, University College of Science, University of Tehran, P.O. Box 14155-6455, Tehran, Iran; 2 Laboratory of Advanced Biotechnologies for Health Assessments (Lab-HA), Lassonde School of Engineering, York University, Toronto, ON M3J 1P3 Canada; 3 Department of Electrical Engineering and Computer Science, Biomedical Engineering Program, York University, 4700 Keele Street, Toronto, ON M3J 1P3 Canada

**Keywords:** Drug analysis, g-C_3_N_4_-CNT nanocomposite, PLS method, FFT-voltametry, Electrochemistry, Biochemistry, Biomarkers, Chemistry

## Abstract

The simultaneous identification of drugs has considerable difficulties due to the intricate interplay of analytes and the interference present in biological matrices. In this study, we introduce an innovative electrochemical sensor that overcomes these hurdles, enabling the precise and simultaneous determination of morphine (MOR), methadone (MET), and uric acid (UA) in urine samples. The sensor harnesses the strategically adapted carbon nanotubes (CNT) modified with graphitic carbon nitride (g-C_3_N_4_) nanosheets to ensure exceptional precision and sensitivity for the targeted analytes. Through systematic optimization of pivotal parameters, we attained accurate and quantitative measurements of the analytes within intricate matrices employing the fast Fourier transform (FFT) voltammetry technique. The sensor’s performance was validated using 17 training and 12 test solutions, employing the widely acclaimed machine learning method, partial least squares (PLS), for predictive modeling. The root mean square error of cross-validation (RMSECV) values for morphine, methadone, and uric acid were significantly low, measuring 0.1827 µM, 0.1951 µM, and 0.1584 µM, respectively, with corresponding root mean square error of prediction (RMSEP) values of 0.1925 µM, 0.2035 µM, and 0.1659 µM. These results showcased the robust resiliency and reliability of our predictive model. Our sensor’s efficacy in real urine samples was demonstrated by the narrow range of relative standard deviation (RSD) values, ranging from 3.71 to 5.26%, and recovery percentages from 96 to 106%. This performance underscores the potential of the sensor for practical and clinical applications, offering precise measurements even in complex and variable biological matrices. The successful integration of g-C_3_N_4_-CNT nanocomposites and the robust PLS method has driven the evolution of sophisticated electrochemical sensors, initiating a transformative era in drug analysis.

## Introduction

Morphine (MOR), a naturally occurring opiate derived from opium, exerts its pharmacological effects by modulating the central nervous system, rendering it an effective agent for alleviating severe pain, particularly in post-surgery patients^1–3^. In contrast, methadone (MET) represents a pure industrial analgesic pharmaceutical that is used for medication-assisted treatment of opioid use disorder. Its heightened lipophilicity enables prolonged retention in the liver and other tissues, yielding a slow distribution profile, consequently endowing MET with an extended duration of action compared to MOR^4,5^. However, it is imperative to acknowledge that concurrent administration of MOR and MET with other medications can lead to central nervous system perturbations and potentiate drug interactions, necessitating cautious management in clinical practice. Given the potentially toxic and lethal effects associated with high doses of MOR and MET, precise and sensitive measurement of their concentrations in patients’ blood or urine becomes paramount^6–8^. The concentration levels of MOR and MET in biological matrices are intrinsically linked to the administered dosages, which for an average individual (70 kg body weight) may vary from 1.5 to 400 mg. These ranges apply to legally prescribed levels of these substances in the body fluids. However, concentrations from illegal or self-administered use can vary widely, potentially reaching lethal doses in biological samples and other body organs. After taking these drugs, the dynamic concentration of these substances in serum exhibits a temporal variation, delineated by a range of 0.25 to 3 µM. In scenarios of toxicological dosing, exceeding 500 mg, concentrations can proliferate to levels surpassing 5 µM^9^. Furthermore, detecting uric acid (UA) as the principal breakdown product of purine metabolism assumes profound significance^10,11^. Anomalous levels of UA and the subsequent deposition of its crystals in joints can give rise to diverse ailments, including but not limited to pneumonia, gout, hyperuricemia, and cardiovascular and renal disorders^12,13^.

Over the past decades, various methodologies have emerged to detect MOR, MET, and UA accurately. These encompass high-performance liquid chromatography (HPLC)^14^, liquid chromatography-mass spectrometry (LC–MS)^15^, spectroscopy^16^, supercritical fluid chromatography^17^, and chemiluminescence^18^. Although these techniques demonstrate commendable sensitivity and remarkable reliability, their practical utility is constrained by certain drawbacks, including time-consuming procedures, the necessity for sample pre-treatment, analytical complexity, and the associated costs and labor intensiveness^19,20^. Consequently, a compelling need arises for an accurate, sensitive, cost-effective, and simplified detection system. In this context, electrochemical methods have garnered attention due to their inherent advantages, rendering them feasible and advantageous for analytical purposes^21,22^. Among these methods, fast Fourier transform square-wave voltammetry (FFT-SWV) is one of the most sensitive techniques for rapid and straightforward trace detection of compounds. The FFT-SWV leverages the power of Fourier transform filtering, in conjunction with electrochemical principles, to effectively mitigate environmental interferences and enhance the detection limit and overall sensitivity^23,24^.

The principle underpinning the simultaneous electrochemical detection of multiple analytes in electrochemical sensors relies on the requirement that electrochemically active species should undergo oxidation at distinct oxidation potentials. However, the challenge arises when two or more analytes exhibit overlapping oxidation potentials in a bare electrode configuration, thus hindering the simultaneous detection of multiple analytes. To overcome this limitation, considerable attention has been directed towards developing various surface-modified electrodes in the past decade^25,26^. Carbon-based nanoparticle-modified electrodes have garnered substantial attention due to their remarkable attributes, encompassing significant catalytic activity, large surface area, high electron transfer rate in electrochemical reactions, remarkable sensitivity and stability, and low detection limits^27–30^. Among them, graphitic carbon nitride (g-C_3_N_4_) with a layered structure is one of the most resilient carbon nitride substances^31,32^. The unique characteristics of g-C_3_N_4_ include high thermal and chemical stability, a distinctive electronic structure resulting from the characteristic sp^2^ hybridization of carbon and nitrogen atoms, abundant surface active sites, and singular optical features. These remarkable properties have applications in diverse fields, including photocatalysis, energy storage and conversion devices, and biological/chemical sensors^33–35^. However, the inherent poor conductivity of bare g-C_3_N_4_ poses a limitation for its direct use in electrochemical sensors. To address this drawback, one viable solution is to combine it with other materials, such as conducting polymers, metal oxides, and carbon-based materials^36,37^. Carbon nanotube (CNT) has emerged as a prominent choice due to its exceptional features, including outstanding chemical and mechanical stability, remarkable electrical conductivity, and high specific surface area.

As a result, CNT has found extensive application across diverse domains like electrocatalysis, photocatalysis, electromagnetic shielding, and electrochemical sensors^38–41^. In a research conducted by Wang and colleagues, an electrode modified with a 3D g-C_3_N_4_/multi-wall (MW) CNTs/graphene oxide (GO) hybrid was fabricated and applied as an electrochemical sensor for the simultaneous detection of uric acid, dopamine, and ascorbic acid^42^. The integration of g-C_3_N_4_ and GO in the hybrid composite led to a strong synergy, while the high conductivity of MWCNTs further enhanced the electrocatalytic capability of the modified electrode. As a result, the modified electrode demonstrated efficiently increased electrocatalytic activity, enabling the oxidation of uric acid, dopamine, and ascorbic acid and facilitating the distinct identification of their respective anodic peaks. In another research by Karimi-Harandi et al.^43^, a carbon paste electrode was modified with a nanocomposite consisting of zeolitic imidazolate framework-8 (ZIF-8), g-C_3_N_4_, and reduced graphene oxide (RGO). This modified electrode was applied as an electrochemical sensor for the highly sensitive simultaneous detection of citalopram and selegiline. The fabricated electrode demonstrated excellent sensing performance, as validated by its low detection limit and broad linear ranges for both drugs.

Adopting a multivariate calibration methodology is an alternative and effective strategy to tackle the challenge of overlapping signals from multiple analytes in simultaneous sensing. This approach entails using full response-profile data to construct a predictive model that can effectively handle the complexities of the analytical matrix. To establish a correlation between the physicochemical characteristics of the analyte and its response profile, both linear and non-linear calibration procedures can be applied as predictive models. Given their compatibility and simplicity, linear calibration methods are often favored when a direct description of physicochemical characteristics is desired. Partial least-squares regression (PLSR) is one of the most widely adopted linear methods for data processing^44^. This approach offers several advantages, making it a popular choice in analytical applications. It effectively utilizes the complete range of response profiles, enabling fast detection of mixture components, mitigating the impact of interface effects, disregarding the concentration of non-selected analytes, and significantly improving overall performance^45–47^.

**Figure 1:**
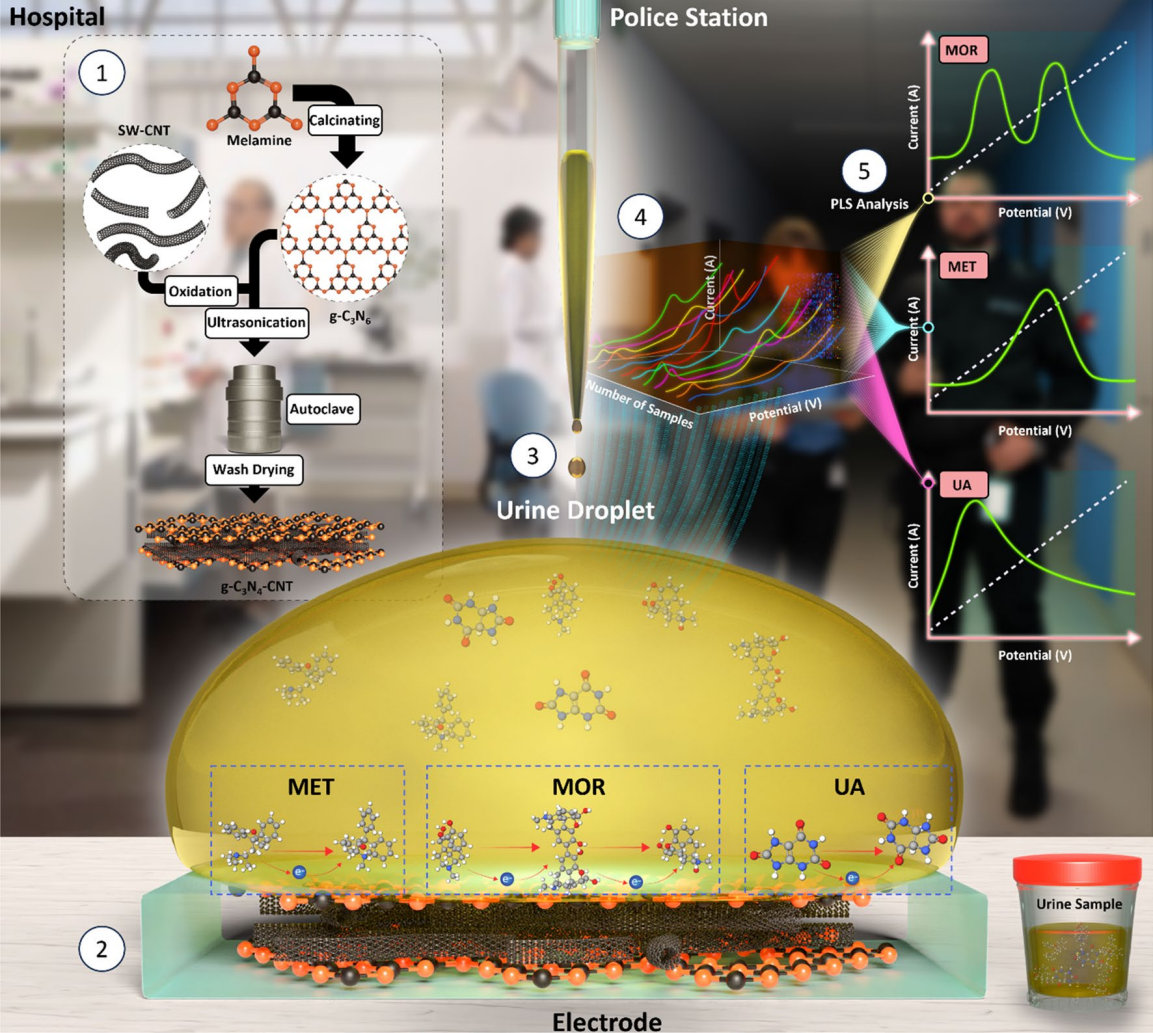
Schematic illustration of the sensing setup for simultaneous detection of morphine (MOR), methadone (MET), and uric acid (UA) in urinary biofluids; (1) synthesis process of single wall carbon nanotubes (SW-CNT) modified with graphitic carbon nitride (g-C_3_N_4_) nanosheets (g-C_3_N_4_-SW-CNT), (2) deposition of the synthesized nanocomposite of g-C_3_N_4_-SW-CNT on the surface of the glassy carbon electrode, (3) the electrochemical reaction of MOR, MET, and UA within the complex matrix of urine at the surface of the electrode, (4) fast Fourier transform (FFT) square-wave voltammograms obtained for samples containing these three analytes at various concentrations, (5) partial least squares (PLS) analysis as multivariate calibration approach for predictive modeling.

In this study, the primary aim revolved around leveraging the advantages of electrochemical methodologies, coupled with electrode surface modification and computational techniques, to develop an electrochemical sensor of utmost proficiency. The objective was to immobilize g-C_3_N_4_-CNT onto a glassy carbon electrode (g-C_3_N_4_-CNT-GCE) that manifests superior sensitivity and selectivity in the simultaneous determination of MOR, MET, and UA. In our prior research, we engineered the glassy carbon electrode via functionalization with CMK-5 ordered mesoporous structure, facilitating the simultaneous quantification of MOR and MET. This modification, employing g-C_3_N_4_-CNT-GCE, yielded a notably enhanced efficacy and stability for concurrent analytical detection objectives^47^. The fabricated sensors were subjected to comprehensive analyses, exploring their structural and electrochemical attributes. Among the various electrochemical techniques employed, FFT-SWV emerged as the most discerning approach, effectively enabling the quantitative assessment of the three targeted analytes. PLS, a widely embraced linear method within the domain of multivariate calibration methodologies, was employed to process the obtained data. The resulting calibration curve was successfully established, facilitating the accurate MOR, MET, and UA detection within real urine samples (Fig. 1).

## Experimental section

### Materials and real urine samples

All chemicals utilized in this study were of reagent grade, and no further purification steps were undertaken before their application. A 1 mM phosphate buffer solution was prepared with di-potassium hydrogen phosphate (99%, 7778-77-0) and potassium dihydrogen phosphate (99%. 7758-11-4), procured from Merck. The pH adjustment was effectively accomplished by employing potassium hydroxide (85%, 1310-58-3) and hydrochloric acid (37%, 7647-01-0). Furthermore, melamine (99%, 108-78-1), Single wall CNT (SWCNT) ($\ge$ 95%, 308068-56-6), sulfuric acid (H_2_SO_4_) (98%, 7664-93-9), hydrogen peroxide (H_2_O_2_) (35%, 7722-84-1), nitric acid (HNO_3_) (65%, 7697-37-2), potassium ferricyanide (K_3_Fe(CN)_6_) (99%,13746-66-2), ascorbic acid (99%, 50-81-7), sodium chloride (NaCl) ($\ge$ 99%, 7647-14-5), potassium chloride (KCl) (≥ 99%, 7447-40-7), aluminum chloride hexahydrate (AlCl_3_.6H_2_O) (99%, 7784-13-6), uric acid (99%, 69-93-2), glucose (99.5%, 50-99-7), sucrose (99.5%, 57-50-1), fructose (99%, 57-48-7), isopropanol (≥ 99%, 67-63-0), copper (II) sulfate pentahydrate (≥ 98%, 7758-99-8), lead (II) nitrate (99%, 10099-74-8), and chromium (III) chloride (99%, 10025-73-7), was sourced from Sigma-Aldrich. Morphine sulfate (99%, 6211-15-0), Methadone hydrochloride (99%, 1095-90-5), and ( ±)-Ibuprofen (99%, 15687-27-1) were acquired from Daropakhsh pharmaceutical company, ensuring the use of premium-grade compounds. Furthermore, 1 mM stock solutions of morphine, methadone, and uric acid were meticulously prepared in the phosphate buffer solution, providing accurate and consistent concentrations for subsequent experimental procedures. The urine samples were obtained from healthy volunteers at Imam Khomeini Hospital. Informed consent was obtained from all subjects and/or their legal guardian(s). All experimental protocols (including the use of urine samples) were approved by the University of Tehran’s Research Ethics Committee, reference number of IR.TUMS.REC.1400.041.

### Synthesis of g-C_3_N_4_ and CNT-COOH and their combination

The synthesis of g-C_3_N_4_ involved a polycondensation process utilizing melamine as its precursor, following a step-by-step procedure^48^. Initially, 10 g of melamine was placed in a crucible and heated in a muffle furnace at a controlled temperature of 550 °C for 4 h, with a gradual temperature increase at 2 °C per minute. The resulting sample was yellow and referred to as g-C_3_N_4_.

CNT-COOH was synthesized in an optimized two-step oxidation method^49^. In the first step, 0.1 g of CNT was placed in a 6 mL H_2_O_2_/H_2_SO_4_ 1:3 (v/v) solution before sonication at 60 °C for 2 h. After oxidation, the resulting mixture was diluted with deionized water, and the solid residue was separated through centrifugation at 4000 rpm. The obtained solid was washed three times with DI water and dried overnight at 50 °C. The oxidized CNT was further functionalized in the next step by adding 6 mL HNO_3_/H_2_SO_4_ 1:3 (v/v) to the solid and placed in an ice bath under stirring. The mixture was sonicated at 60 °C to achieve a homogenized suspension. Afterward, the suspension was centrifuged at 4000 rpm, and the solid was washed with deionized water until it reached a pH of 7. Finally, the as-prepared CNT-COOH sample was dried in a vacuum oven for 24 h at 65 °C to ensure complete drying and stabilization. Throughout the text, CNT-COOH is abbreviated as CNT.

To prepare the nanocomposite of g-C_3_N_4_-CNT; first, a suspension of 5 mL containing 1 mg/mL of CNT was mixed with another 5 mL suspension containing 1 mg/mL of g-C_3_N_4_. To achieve homogeneity, 10 mL of isopropanol was added to this mixture before subjecting it to sonication. Subsequently, the homogenized suspension was transferred to a 40 mL stainless steel autoclave. Inside the autoclave, the temperature was gradually increased at 2 °C per minute until reaching 120 °C, where it was maintained for 48 h. After the solvothermal treatment, the resulting nanocomposite samples were washed three times with DI water and dried in a vacuum oven at 80 °C for 12 h. This process facilitated the transformation of g-C_3_N_4_ and CNT into the desired g-C_3_N_4_-CNT nanocomposite through a solvothermal self-assembly process^49^, as illustrated in Fig. S1. Subsequently, a suspension of g-C_3_N_4_-CNT with a concentration of 3 mg/mL in water was subjected to sonication for 15 min to achieve a homogenous suspension. Finally, a precise volume of 2 µL of the suspension was evenly deposited onto the surface of the GC electrode. The deposited suspension was then dried under an infrared (IR) lamp.

### Electrochemical characterization

All electrochemical experiments were conducted using a μStat-i 400s portable Potentiostat/Galvanostat/Impedance Analyzer (Metrohm Dropsens, Netherlands), controlled by a personal computer equipped with DropView 8400 software. The device features a three-electrode system comprising a 2 mm diameter glassy carbon electrode (GCE) as the working electrode, a platinum wire as the auxiliary electrode, and an Ag/AgCl electrode as the reference electrode. Electrochemical Impedance Spectroscopy (EIS) was conducted within a solution comprising 5 mM Potassium ferricyanide and 0.1 M potassium chloride, totaling 10 mL in volume, by applying a frequency from 100 mHz to 100 kHz. The applied DC potential was 50 mV, and the applied AC potential amplitude was 10 mV. The quantitative electrochemical assessment of MOR, MET, and UA was executed utilizing a custom-designed Autolab electrochemical system equipped with a three-electrode cell configuration. FFT-SWV is an advanced electrochemical technique derived from the SWV method, employing the discrete FFT approach for background subtraction and two-dimensional integration of the electrode response to analyze chemical compounds. Detailed insights into this methodology can be accessed through Fig. S5, along with the corresponding explanation provided in the Supplementary Information (SI) file under the section labeled SI-S2.

### Structural characterization

X-ray photoelectron spectroscopy (XPS) with a monochromatic A1 Kα radiation source was employed to inspect the valence of the elements as well as their composition. Transmission electron microscopy (TEM) and high-resolution transmission electron microscopy (HRTEM) were executed on a Philips CM30 200 kV and HR-TEM FEI TECNAI F20 instrument. Scanning electron microscopy (SEM) equipped with TeScan-Mira III EDX system and field emission scanning electron microscopy (FESEM) was utilized for morphological and elemental analysis. The UV–vis diffuse reflectance spectroscopy (DRS) (Scinco 4100) characterized the optical properties of the synthesized materials. Photoluminescence (PL) spectra were obtained by Perkin Elmer LS55 luminescence. A Metrohm 744 pH meter was employed for pH adjustments, and an Ultrasonic cleaner was used to homogenize the samples.

### Multivariate calibration

The PLS method is a statistical technique used for building predictive models between two sets of variables, usually denoted as X and Y. It is beneficial when there are many correlated predictor variables and works by reducing the dimensionality of the X space and finding the relationship with the Y space.

The basic formula for the PLS algorithm can be represented as:

1 $${\text{Y}}={\text{XB}}+{\text{E}},$$

where Y is the matrix of dependent variables (responses or outputs), X is the matrix of independent variables (predictors or inputs), B is the matrix of regression coefficients that describes the relationship between X and Y, and E is the matrix of residuals or errors (the difference between the observed and predicted values). For further elucidation of the PLS method, additional details are accessible in SI-S3.

## Results and discussion

### Surface characterization of g-C_3_N_4_-CNT

**Figure 2:**
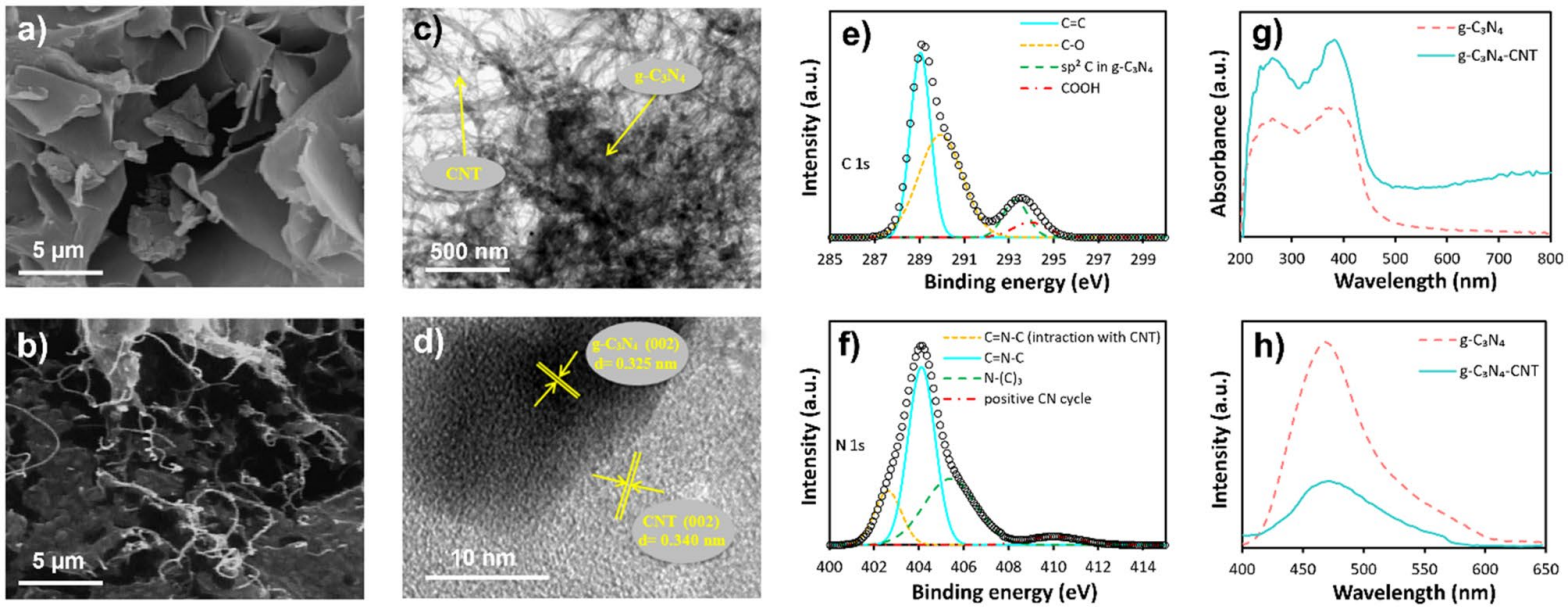
Physical characterization of graphitic carbon nitride (g-C_3_N_4_) nanosheets and g-C_3_N_4_ modified with carbon nanotubes (CNTs). (**a**) Field emission scanning electron microscopy (FESEM) images of g-C_3_N_4_ and (**b**) g-C_3_N_4_-CNT composite. (**c**) Transmission electron microscopy (TEM) and (**d**) high-resolution TEM (HRTEM) image of g-C_3_N_4_-CNT composite. X-ray photoelectron spectroscopy (XPS) spectra of the (**e**) carbon and (**f**) nitrogen elements in the g-C_3_N_4_-CNT composite. (**g**) UV–vis spectrum and (**h**) fluorescence spectrum related to g-C_3_N_4_ and g-C_3_N_4_-CNT.

The morphology analysis of g-C_3_N_4_ and g-C_3_N_4_-CNT nanocomposite was conducted using FESEM and HRTEM. In the FESEM image of g-C_3_N_4_ (Fig. 2a), a plate-like structure with a thickness of less than 100 nm is prominently visible. Transitioning to the FESEM image of g-C_3_N_4_-CNT (Fig. 2b), the placement of CNTs amidst the g-C_3_N_4_ sheets becomes evident, confirming the successful synthesis of the composite. The TEM image of the g-C_3_N_4_-CNT composite depicted in Fig. 2c illustrates the homogeneous distribution of CNTs within the g-C_3_N_4_ sheets. Furthermore, Fig. 2d presents the HRTEM image, revealing distinct crystal planes of g-C_3_N_4_ (002) and CNT (002). The corresponding lattice spacing for these planes is measured at 0.325 and 0.340 nm, respectively, under previous findings^50,51^. The HRTEM image underscores a compatible interaction between g-C_3_N_4_ and CNT, forming the g-C_3_N_4_-CNT composite. The pronounced quality of the composite synthesis is evidenced by the clarity observed in the HRTEM image.

Regarding elemental analysis, the Energy-dispersive X-ray (EDX) spectrum of the g-C_3_N_4_-CNT composite has been presented and can be observed in Fig. S2a. Within this EDX spectrum, the presence of three distinct peaks corresponding to carbon, nitrogen, and oxygen is clearly discernible within the nanocomposite. Notably, the relative sizes of these peaks reveal the qualitative distribution of carbon, nitrogen, and oxygen elements within the synthesized composite. The relatively prominent carbon peak compared to the nitrogen peak and the nitrogen peak compared to the oxygen peak provide qualitative insights into the elemental ratios present within the composite structure. Furthermore, in order to comprehensively elucidate the interplay between CNT and g-C_3_N_4_, the XPS spectrum of the g-C_3_N_4_-CNT composite was procured. The collective XPS spectrum of this composite is illustrated in Fig. S2b, manifesting the distinct presence of carbon, nitrogen, and oxygen elements. Evident peaks emerge at binding energies of 289.5 eV, 404.7 eV, and 537.7 eV, corresponding to C1s, N1s, and O1s, respectively. More intricate scrutiny is warranted; thus, the deconvoluted peaks of the carbon and nitrogen components are delineated in Fig. 2e and f. Within the C1s spectrum, four distinct peaks are localized at binding energies of 289.1 eV, 290.1 eV, 293.4 eV, and 294.1 eV, denoting the C = C bond, C-O bond, sp^2^ carbon in g-C_3_N_4_, and COOH-carbon, respectively^52^. Delving into the N1s deconvolution, a quartet of peaks emerges. The peak situated at 402.7 eV corresponds to N atoms (sp^2^ bond) within triazine rings (C–N=C), indicating an interaction with CNT.

Similarly, the peak at 404.1 eV signifies sp^2^-bonded N atoms within triazine rings (C–N=C). The third peak, at 405.4 eV, is attributable to N atoms in N–(C)_3_. Lastly, a minor intensity peak at 410.1 eV signifies positively charged CN heterocycles and cyano groups, a result of protonation of g-C_3_N_4_ through interaction with carboxylic acid groups in CNT^53^.

These findings substantiate a robust interaction between g-C_3_N_4_ and CNT within the g-C_3_N_4_-CNT composite. This interaction manifests along two distinct pathways: the first involves π-π interactions between the conjugated triazine group in g-C_3_N_4_ and the graphene units in CNT, while the second stems from electrostatic interactions between COO groups of CNT and the positive charge on g-C_3_N_4_^52^.

The assessment of the absorption spectra for both g-C_3_N_4_ and g-C_3_N_4_-CNT samples was conducted by UV–V is spectroscopy. Illustrated in Fig. 2g, the spectrum of g-C_3_N_4_ unveils two strong absorption peaks at 264 nm and 380 nm, allocated to π → π* and n → π* electronic transitions, correspondingly^54^. Analogously, the absorption profile of the g-C_3_N_4_-CNT nanocomposite exhibits two peaks akin to those of g-C_3_N_4_; however, the absorption intensity of the nanocomposite is augmented. This heightened absorption intensity can be rationalized through the π-π interaction between g-C_3_N_4_ and CNT, contributing to this enhancement^49^. Fluorescence spectroscopy is An alternative analytical approach to probe the bond formation between g-C_3_N_4_ and CNT. Illustrated in Fig. 2h, g-C_3_N_4_ demonstrates a robust emission peak at 470 nm. Upon the incorporation of CNT into g-C_3_N_4_, resulting in the g-C_3_N_4_-CNT nanocomposite, the intensity of fluorescence emission compared to g-C_3_N_4_ decreased significantly. This phenomenon underpins the significant π-π stacking interaction between g-C_3_N_4_ and CNT. This interaction contributes to shifting the Highest Occupied Molecular Orbital (HOMO) level of g-C_3_N_4_ to lower energy levels, leading to the quenching of fluorescence^49,55^.

### Evaluation of electrochemical properties through EIS and cyclic voltammetry (CV)

EIS was employed to determine the electron transfer rates across distinct modified electrodes. The resistance that electrons show against transfer (R_et_) is derived from the dielectric and insulating properties of the electrode/electrolyte interface^56^. The Nyquist diagram in Fig. S3 illustrates the GCE, CNT-GCE, and g-C_3_N_4_-CNT-GCE configurations within a 5 mM ferrocyanide and 0.1 M potassium chloride solution. For the unmodified GCE, R_et_ measured at approximately 301.17 Ω. Upon introducing a coating of CNT onto the electrode surface, R_et_ declined to around 98.32 Ω. Finally, with the electrode modified with g-C_3_N_4_-CNT, the R_et_ value further diminished to 86.31 Ω—significantly lower than the other two electrodes. The insights drawn from the EIS results suggest that the successful electrode coating involved a composite with heightened conductivity and decreased electron transfer resistance.

**Figure 3:**
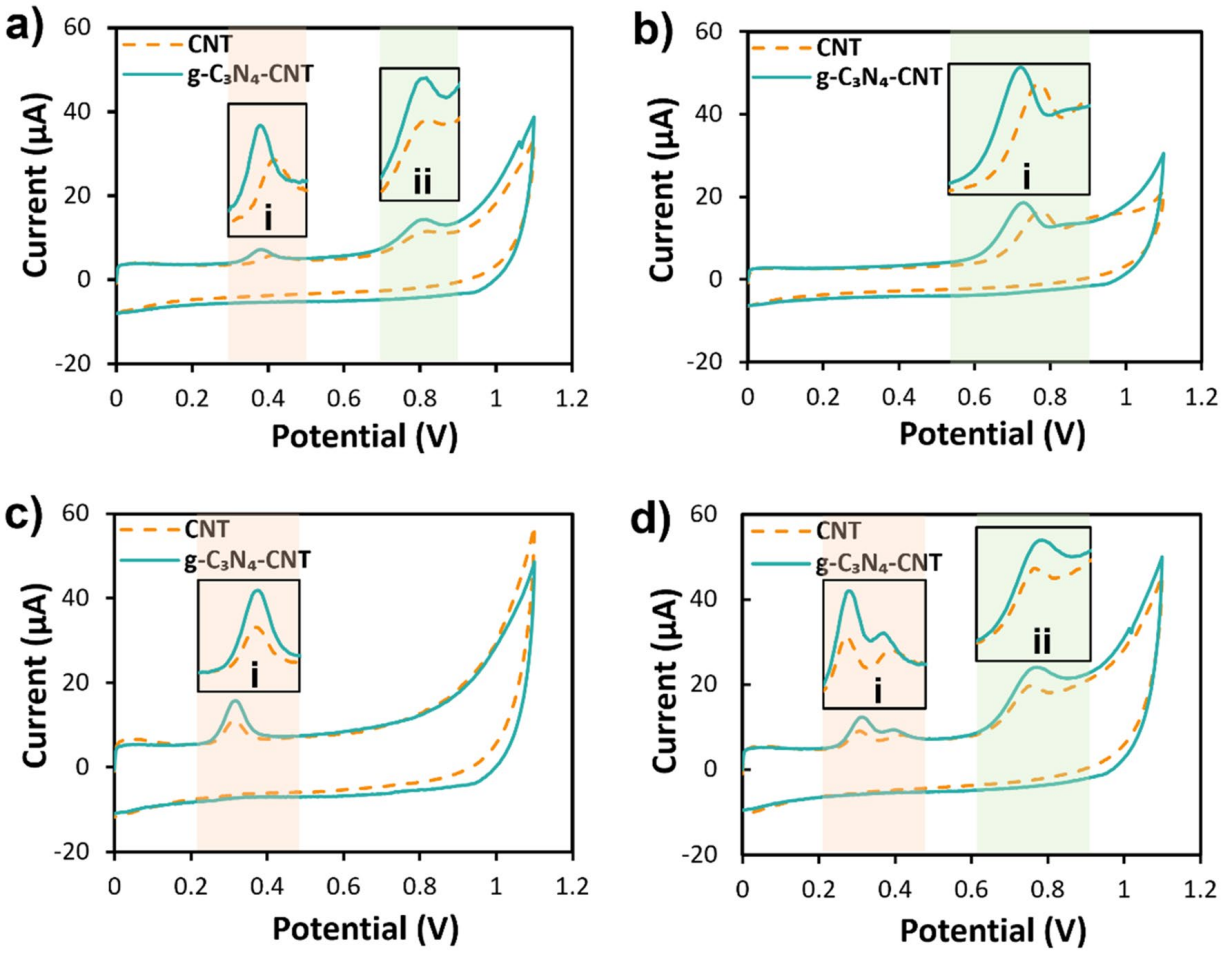
Electrochemical characterization of CNT-GCE and g-C_3_N_4_-CNT-GCE, both in the presence of individual biomolecules and under simultaneous exposure. Cyclic voltammetry diagrams for CNT-GCE and g-C_3_N_4_-CNT-GCE in the presence of (**a**) MOR, indication 2 peaks (i and ii), (**b**) MET with one peak (i), (**c**) UA with one peak (i), and (**d**) a mixture of MOR, MET, and UA at 5 μM concentration.

CV was employed to investigate the electrochemical behavior of MOR, MET, and UA on the surfaces of CNT-GCE and g-C_3_N_4_-CNT-GCE electrodes. The cyclic voltammograms of fabricated electrodes were obtained in a 0.1 M phosphate buffer solution (pH of 8) at the scan rate of 0.1 V/s, with the potential range spanning from 0 to 1.1 V. The CV profile of the buffer blank system in the presence of g-C_3_N_4_-CNT-GCE is shown in Fig. S4. These results confirm the absence of electrochemical peaks in the absence of analytes. Figure 3a–d illustrate the CV profiles of CNT-GCE and g-C_3_N_4_-CNT-GCE in the presence of 5 μM MOR, MET, and UA. These figures show that anodic peaks manifest for both the modified electrodes. The anodic peaks corresponding to MOR, i and ii, on the CNT-GCE, emerge at the potentials of 0.42 and 0.83 V. At the same time, on the g-C_3_N_4_-CNT-GCE, they occur at slightly lower potentials of 0.39 and 0.81 V and higher peak currents compared to those on the CNT-GCE.

The observed shift of the anodic peaks of MOR towards more negative potentials accompanied by the augmented current in the presence of the g-C_3_N_4_-CNT-GCE relative to CNT-GCE, underscore the electrocatalytic traits of the g-C_3_N_4_-CNT composite in the electrochemical quantification of MOR. Figure 3b’s MET analysis shows distinct peaks at 0.79 V for CNT-GCE and 0.73 V for g-C_3_N_4_-CNT-GCE, with the latter exhibiting increased peak currents due to improved electrocatalytic properties. Figure 3c’s UA analysis reveals peaks at around 0.33 V for CNT-GCE and 0.34 V for g-C_3_N_4_-CNT-GCE, with a notable current increase in the latter, attributed to its higher conductivity and electrocatalytic efficiency. Concluding this analysis, the simultaneous presence of MOR, MET, and UA was examined using both electrode configurations, as indicated in Fig. 3d. Significantly, the observed overlap between the anodic peak of UA and the initial anodic peak of MOR (i) on the g-C_3_N_4_-CNT-GCE is more pronounced than on the CNT-GCE. This phenomenon is attributed to the significant shift of the first anodic peak of MOR towards more negative potentials, relative to UA, in the presence of the g-C_3_N_4_-CNT composite.

Conversely, the enhancement observed in the anodic current for both UA and MOR stems from including the g-C_3_N_4_-CNT composite. The capacity to segregate these compounds can be achieved based on the extent of overlapping and the increase in current brought about by multivariable calibration methodologies. Moreover, the second anodic peak (ii) of MOR and the electrochemical peak corresponding to MET exhibit a pronounced overlap in the presence of both electrode configurations. Nonetheless, it should be noted that when the g-C_3_N_4_-CNT-GCE is utilized, the composite results in a broader peak width for the MOR and MET mixture compared to the CNT-GCE.

It is analyzing the single-component curves presented in Fig. 3a and b, it is evident that the potential discrepancy between the second MOR peak and the anodic peak of MET is 80 mV in the presence of the g-C_3_N_4_-CNT-GCE, while it stands at 40 mV in the presence of the CNT-GCE. These observations indicate a reduced overlap of the two anodic peaks when utilizing the g-C_3_N_4_-CNT composite.

### Optimization of pH, accumulation time, and FFT-SWV variables

**Figure 4:**
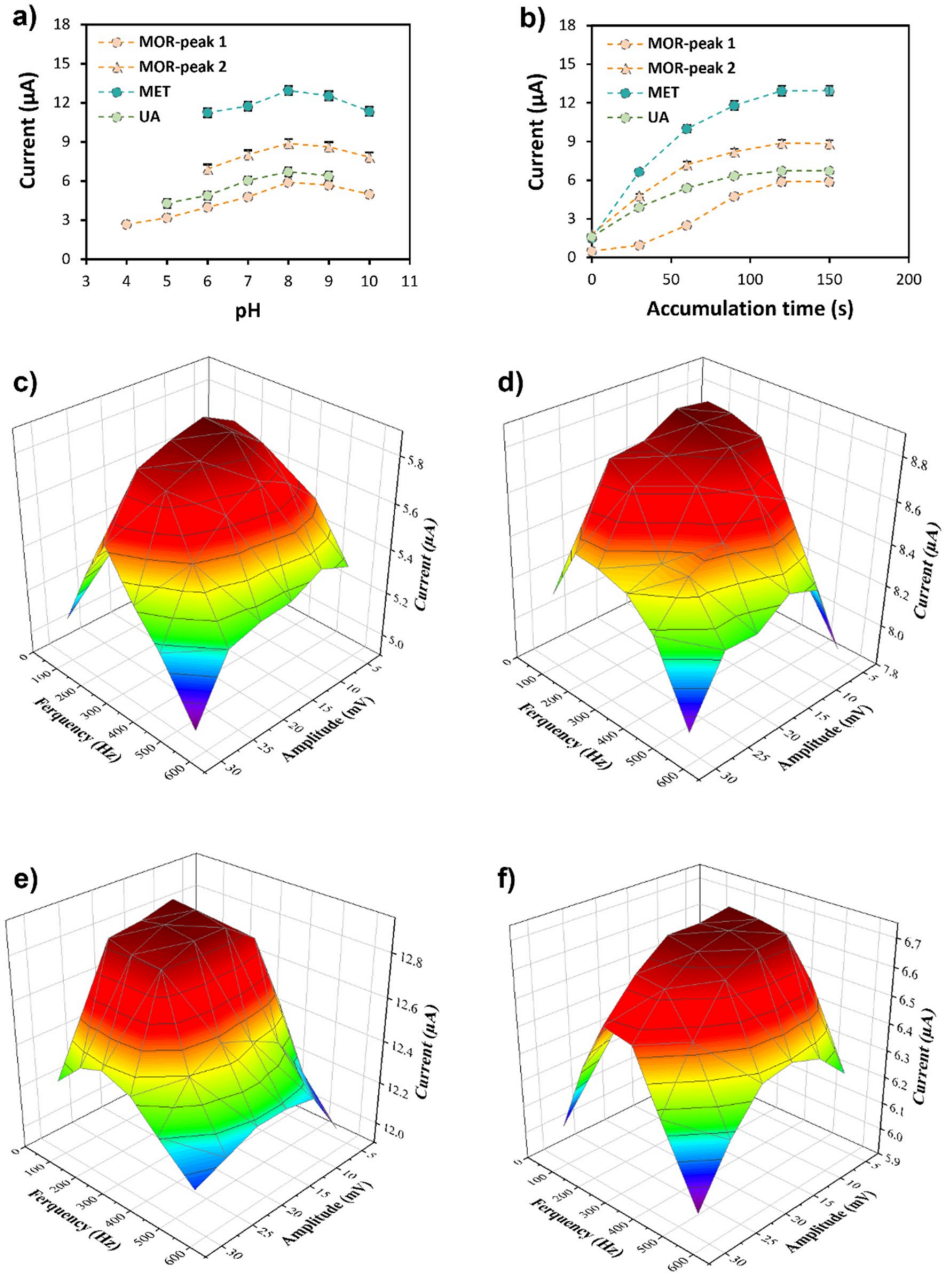
Optimization of specific parameters affecting the efficiency and operational performance of the g-C_3_N_4_-CNT sensor. (**a**) pH optimization of MOR, MET, and UA. (**b**) The effect of accumulation time on the electrochemical response of MOR, MET, and UA at 5 μM concentration of the analytes individually (n = 5). Optimization of frequency and amplitude in fast Fourier transform square-wave voltammetry (FFT-SWV) technique for the (**c**) first peak of MOR, (**d**) second peak of MOR, (**e**) MET, and (**f**) UA electrochemical response at 5 µM concentration and pH = 8.

The pH of the measurement solution stands as a critical determinant in electrochemical analyses, primarily due to the influential roles that groups containing ${OH}^{-}$ and ${H}^{+}$ play in most electrochemical reactions. The effect of pH on the current of the electrochemical reactions of MOR, MET, and UA was assessed at a concentration of 5 µM in phosphate buffer. As depicted in Fig. 4a, MOR, MET and UA peaks exhibited an increase in current with rising pH values to 8. However, at pH values of 9 and 10, a slight decrease in the responses was observed. Also, a significant response was not observed for the MET and second peak of MOR at pH less than 6, UA at pH less than 5, and the first peak of MOR at pH less than 4. Conclusively, a pH of 8 was determined for further experiments, as it yielded the maximum response.

To explore the effect of accumulation on the efficiency of the electrochemical measurements of MOR, MET, and UA, a 5 µM solution of the samples was subjected to analysis (Fig. 4b). The measurements were conducted in 30 s intervals within 0 to 150 s. Fluctuations in the currents of MOR and MET persisted until 120 s, whereas it sustained the same quantity up until 150 s. In the case of UA, variations endured until 90 s before stabilizing. Consequently, the optimum measurement time of 120 s was selected for MOR, MET, and UA analyses.

Amplitude and frequency constitute pivotal parameters within the framework of FFT-SWV. Amplitude signifies the magnitude of potential disparity between the maximal positive and negative values within the square-wave, a parameter critically involved in determining sensitivity and resolution in the FFT-SWV method. Amplifying the amplitude generally enhances the sensitivity and peak current of the voltammetric response, as it provides a greater driving force for redox reactions at the electrode interface. However, excessive amplification could lead to elevated background currents and noise, potentially reducing signal-to-noise ratio and impeding resolution. Striking the optimal balance between sensitivity and resolution mandates judicious selection of amplitude.

Frequency influences the analysis speed and the diffusional transport of analytes to the electrode surface. Elevating frequency quickens analysis by completing more potential cycles in a shorter span. Higher frequencies can also mitigate capacitive background currents, boosting signal-to-noise ratio and lowering detection limits. Nevertheless, extremely high frequencies might curtail the diffusion time of analytes to the electrode, causing reduced peak currents and sensitivity. The choice of optimal frequency hinges on the specific system under scrutiny and the desired trade-off between speed, sensitivity, and signal-to-noise ratio. As a result, amplitude and frequency profoundly influenced square-wave voltammetry, with their optimal values contingent upon the specific electrochemical system. The meticulous tuning of these parameters is imperative for maximizing the FFT-SWV technique’s sensitivity, resolution, and speed.

Figure 4c–f pertains to the investigation of frequency and amplitude influence on the initial electrochemical peak of MOR. It’s evident that within the frequency range of 88 to 256 Hz and the potential range of 5 to 15 mV, the electrochemical response of the first peak’s current demonstrated an upward trend. Subsequently, the response remained relatively steady from the frequency of 256 to 354 Hz and the range of 15 to 25 mV. Beyond a frequency of 354 to 564 Hz and a potential range of 25 to 30 mV, a diminishing trend emerged. Figure 4d and e delve into the impact of altering amplitude and frequency on the second electrochemical peak of MOR and the electrochemical peak of MET. These figures show that both peaks’ electrochemical responses increased within the frequency range of 88 to 177 Hz and the potential range of 5 to 15 mV. The response stabilized within the frequency range of 177 to 256 Hz and the potential range of 15 to 25 mV, followed by a decline until reaching a frequency of 564 Hz and a potential range of 30, likely attributed to slower electrochemical reaction kinetics. Additionally, as depicted in Fig. 4f, the electrochemical peak current of UA escalated with increasing frequency from 88 to 256 Hz and amplitude from 5 to 10 mV. The response remained consistent between the frequency of 256 to 443 and the amplitude of 10 to 20 mV, followed by a subsequent decline. Based on these findings, the frequency of 256 Hz and amplitude of 20 mV were identified as the optimal values for these two parameters in implementing the FFT-SWV technique.

### Simultaneous measurement of MOR, MET, and UA

**Figure 5:**
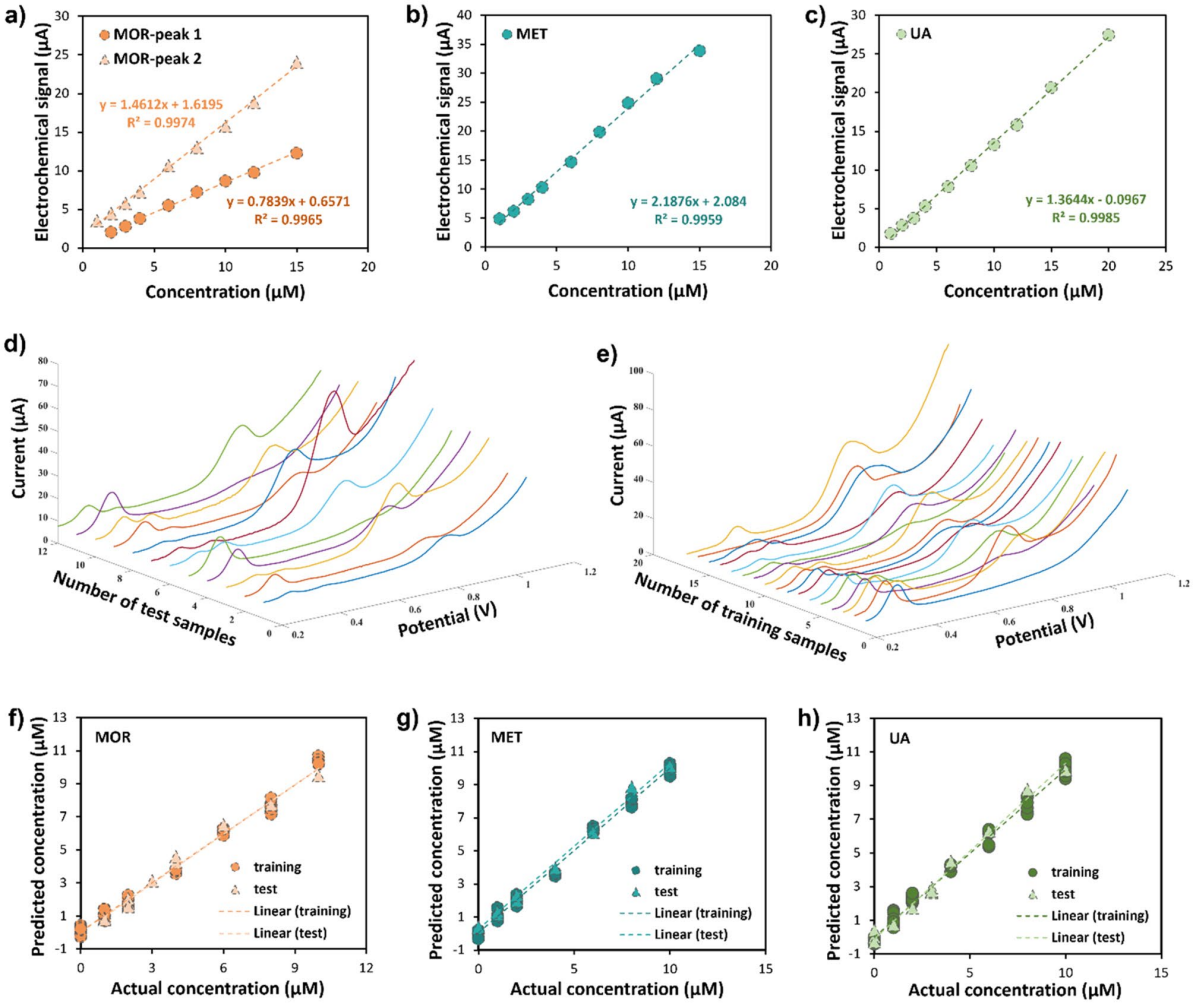
Individual and simultaneous measurement of the target biomolecules. Univariate calibration of (**a**) MOR, (**b**) MET, and (**c**) UA. FFT-SWV voltammogram of the MOR, MET, and UA mixed solutions at various concentrations conducted at a frequency of 256 Hz and an amplitude of 20 mV for the (**d**) test and (**e**) training solutions. Results of calibration and validation of (**f**) MOR, (**g**) MET, and (**h**) UA by the partial least-squares (PLS) model. All 'training' points were repeated five times, and certain 'test' points were repeated three times.

To enhance a model for the simultaneous analysis of MOR, MET, and UA by the PLS method and to achieve a comprehensive understanding of the linear range of the g-C_3_N_4_-CNT-GCE electrochemical sensor, individual univariate calibrations were conducted for each compound (Fig. 5a–c). The resulting analytical information, including the dynamic linear range (DLR), the limit of detection (LOD), and the limit of quantitation (LOQ) are presented in Table 1. The LOD and LOQ follow equations 3S_b_/m and 10S_b_/m, respectively, in which S_b_ is the blank standard deviation and m is the slope of the calibration curve.

**Table 1:** Regression equations, calibration coefficient, limit of detection, and dynamic linear range of g-C_3_N_4_-CNT-GCE electrode for individual detection of MOR, MET, and UA.

Analyte	Calibration equation	R^2^	LOD (µM)	LOQ (µM)	LDR (µM)
MOR peak (1)	y = 0.7839x + 0.6571	0.9965	0.46	1.51	1.5–15
MOR peak (2)	y = 1.4612x + 1.6195	0.9974	0.29	0.95	1–15
MET	y = 2.1876x + 2.084	0.9959	0.25	0.82	1–15
UA	y = 1.3644 x—0.097	0.9985	0.30	1.00	1–20

Upon optimizing the operational parameters of the developed sensor and determining the analytical parameters via univariate calibration, a set of mixing solutions, comprising 17 training samples and 12 test samples, was selected and prepared (Table S1). Test and training solutions responses were recorded using the FFT-SWV technique. Subsequently, voltammograms, as depicted in Fig. 5d and e, were generated to serve as input data for PLS modeling. The model’s efficiency was evaluated across different latent variables (LVs), and ultimately, the first four LVs were deemed to represent the optimal condition for the PLS modeling process.

Table S2 presents the root mean square errors of calibration (RMSEC) for MOR, MET, and UA, which stand at 0.1409, 0.1517, and 0.1143, respectively. The cross-validation procedure was conducted through the leave-one-out method, resulting in root mean square errors of cross-validation (RMSECV) for MOR, MET, and UA as 0.1825, 0.1951, and 0.1584, respectively. To validate the performance of the obtained multivariate calibration model for MOR, MET, and UA, a set of 10 test solutions was evaluated. These solutions encompassed diverse concentrations with the linear concentration range of the three analytes. The root mean squared error of prediction (RMSEP) values were calculated, yielding values of 0.1925, 0.2035, and 0.1659 for MOR, MET, and UA, respectively. Furthermore, the determination coefficient (R_p_^2^) values for the prediction of MOR, MET, and UA were found to be 0.9632, 0.9545, and 0.9651, respectively. This variation in the performance of the model is attributed to the optimization of modeling for UA when compared to MOR, and, similarly, the enhanced modeling of MOR in comparison to MET. This differentiation is related to the minimal perturbation of the UA peak and the first peak of MOR.

The results of quantifying MOR, MET, and UA within both training and test datasets using PLS are depicted in Fig. 5f–h, respectively. These figures indicate reasonable agreement between the predicted and actual concentration levels of MOR, MET, and UA. A comparative analysis of the current approach against techniques delineated in the prior literature is provided in Table S3.

### Interference study

In order to explore potential interferences within the concurrent quantification of MOR, MET, and UA within the intricate matrix of urine, 5 µM solutions of the analytes were employed under optimal conditions. The typical concentrations of some of these potential interferences in urine are as follows: Na^+^ approximately 20,000 µM, K^+^ within the range of 3,500 to 5,000 µM, Cu^2+^ varying from 0.315 to 0.787 μM, Cr^2+^ less than 0.002 μM, Glucose spanning from 0 to 800 µM, and ascorbic acid ranging from 280 to 560 µM. The tolerance threshold for each constituent was predetermined to entail a maximum ± 5% relative predictive deviation. This data demonstrate the ratio of the maximum concentration of interfering species to the concentration of the analyte, represented as Ci/Ca (Table 2). Evidently discernible from the tabulated outcomes is the absence of interference from commonly encountered cationic species during the simultaneous assessment of the analytes. Similarly, saccharine compounds encompassing glucose, sucrose, and fructose exhibited negligible impact, potentially attributed to their constrained permeability through nano-scale apertures and hindered mobility within an electrically charged milieu. Ubiquitous vitamin molecules and psychoactive agents, notably ibuprofen, ascorbic acid, and amphetamine, highly likely to be present within the urine matrix, exhibited negligible perturbation to the FFT-SWV response curve. Foremost among the interfering agents was ascorbic acid, which substantially affected MOR and UA measurements and was attributable to its active participation in electrochemical reactions upon the electrode’s surface. Despite such instances, the sensor conspicuously demonstrated commendable selectivity across the comprehensive evaluation of all three analytes.

**Table 2:** Threshold concentrations ratios of diverse interfering species to 5 µM MOR, MET, and UA solution within a 0.1 M phosphate buffer (pH = 8).

Species	MOR	MET	UA
${Na}^{+}$,${K}^{+}$,${Al}^{3+}$	> 1000	> 1000	1000
${Cu}^{2+}$, ${Cr}^{2+}$, ${Pb}^{2+}$, ${NO}_{3}^{-}$,${SO}_{4}^{2-}$	500	500	500
Glucose, sucrose, fructose	500	500	600
Ibuprofen	350	350	450
Amphetamine	300	300	400
Ascorbic acid	100	150	50

### Reusability and the life-time of the electrochemical sensor

The reusability potential of the electrochemical sensor based on g-C_3_N_4_-CNT was evaluated over 12 iterative trials via the FFT-SWV methodology, employing a 5 µM solution of MOR, MET, and UA within a pH = 8 phosphate buffered saline electrolyte solution. After each measurement, the sensor was thoroughly rinsed using deionized water. The assessable metric for reusability encompassed PLS’s relative predictive error, gauging the fidelity of the sensor’s performance. Figure S6 depicts the FFT-SWV voltammogram, illustrating the results of the 12 measurement iterations for the analytes. PLS-based relative prediction errors for MOR, MET, and UA after completing 12 experiments were found to be 4.2%, 4.9%, and 3.7%, respectively. In fact, the reported data show the percentage of decrease in prediction accuracy. These findings firmly establish the high reproducibility of the sensor’s measurements for MOR, MET, and UA, thus underscoring its superior capability for repeated use. To assess the sensor's life-time, we conducted MET concentration detection tests (targeting 5 µM MET) over seven consecutive days. The results indicated a modest 6.3% reduction in sensor response on the seventh day compared to the first day. These findings are depicted in Fig. S7.

### Quantification of MOR, MET, and UA in authentic urine samples

The experimental protocol for determining MOR, MET, and UA in urine comprised the following steps: Initially, three urine samples were acquired from healthy volunteers, and each sample was subsequently bifurcated into two aliquots. The primary aliquot was subjected to the prescribed conditions as outlined below. To mitigate the presence of proteins within the urine, 20 µL of a 5 M HCl solution was incorporated into the samples, followed by a 0.2 µm filter filtration. For interference minimization and pH stabilization, a 30-fold dilution was applied to the samples using a phosphate buffer solution at pH 8, with subsequent pH adjustment to 8. This primary set of samples was prepared to establish baseline uric acid levels before introducing the analytes.

Specific quantities of MOR, MET, and UA were introduced for the secondary aliquot. To eliminate proteinaceous components and macromolecules from the urine, the samples were prepared under conditions akin to those of the primary aliquots, followed by analogous dilution procedures. Employing the proposed electrochemical sensor, the direct quantification of MOR, MET, and UA in actual urine samples was investigated, employing the standard addition methodology to minimize the matrix effect. A standard solution of 5 µM from each analytes including MOR, MET, and UA was prepared. These standard solutions were then added to four volumetric flasks (VF), with different volumes of buffer added to each (ranging from 0 to VF1, 5 to VF2, 10 to VF3, and 20 to VF4). Subsequently, the solutions in each VF were diluted to 25 mL. Subsequently, the model derived from PLS was applied to determine the concentrations using the diagram obtained from the FFT-SWV technique. A calibration curve was drawn, and the concentration of the real sample was obtained by extrapolating this curve. The resultant concentrations obtained are tabulated in Table 3. Notably, each sample underwent five measurement iterations, yielding relative standard deviation (RSD) values and recovery percentages ranging from 3.71% to 5.26% and 96% to 106%, respectively.

**Table 3:** Recovery studies by PLS multivariable analysis of real samples in the presence of MOR and MET.

Samples	Component	Found before added (µM)	Added (µM)	Found after added (µM)	Recovery (%)	SD (%)	RSD (%)
Urine#1	MOR	0.11	45	43.4	96.5	± 1.75	4.39
	MET	− 0.13	40	41.3	103.3	± 2.17	5.26
	UA	180.8	10	188.9	99	± 8.14	4.31
Urine#2	MOR	− 0.05	30	31.8	106	± 1.18	3.71
	MET	0.15	35	36.4	104	± 1.55	4.27
	UA	200.6	15	217.7	101	± 10.97	5.04
Urine#3	MOR	0.13	35	33.6	96	± 1.38	4.12
	MET	− 0.08	45	44.3	98.4	± 1.73	3.91
	UA	230.41	15	250.8	102.2	± 10.85	4.33

## Conclusions

This study sought to concurrently assess the presence of morphine, methadone, and uric acid utilizing a sensor based on a composite material composed of g-C_3_N_4_ and CNTs integrated into a glassy carbon electrode. The g-C_3_N_4_-CNT composite was synthesized employing the solvothermal technique, incorporating functionalized CNTs. Characterization via electronic microscopies, XPS, UV–vis spectroscopy, and fluorescence analysis elucidated the composite’s structure, revealing a stacking arrangement facilitated by π-π and electrostatic interactions between the CNTs and g-C_3_N_4_ components. Subsequent investigations involved immobilizing and stabilizing the g-C_3_N_4_-CNT composite onto the GC electrode, leading to enhanced charge transfer and electrocatalytic performance compared to pristine CNTs in the electrooxidation processes of morphine, methadone, and uric acid. FFT-SWV, a highly sensitive electrochemical method, was employed for precise quantitative analysis of these analytes. This technique, relying on rapid Fourier transformation and noise reduction through consistent frequency application, demonstrated notably improved sensitivity. Initial assessments of each analyte using the g-C_3_N_4_-CNT-GCE platform showcased predictable linear response ranges and detection limits comparable to conventional electrochemical analysis methods. Subsequently, the challenge of overlapping signals from these analytes was addressed through partial least squares regression, effectively disentangling their signals. Validation solutions confirmed minimal errors in the predictive model, which exhibited high efficacy in analyzing real-world samples. The potential of advancing this research lies in utilizing novel materials to further enhance the composite’s performance and exploring non-linear multivariate calibration techniques to broaden the linear quantification range.

### Supplementary Information

Supplementary Information.

## Data Availability

The datasets used and/or analyzed during the current study are available from the corresponding authors upon reasonable request.

## References

[1] Rajaei M, Foroughi MM, Jahani S, Zandi MS, Nadiki HH (2019). Sensitive detection of morphine in the presence of dopamine with La3+ doped fern-like CuO nanoleaves/MWCNTs modified carbon paste electrode. J. Mol. Liq..

[2] Akbari S, Jahani S, Foroughi MM, Nadiki HH (2020). Simultaneous determination of methadone and morphine at a modified electrode with 3D β-MnO_2_ nanoflowers: Application for pharmaceutical sample analysis. RSC Adv..

[3] Liu L (2021). Carboxyl-fentanyl detection using optical fibre grating-based sensors functionalised with molecularly imprinted nanoparticles. Biosens. Bioelectron..

[4] Khorablou Z, Shahdost-Fard F, Razmi H (2021). Flexible and highly sensitive methadone sensor based on gold nanoparticles/polythiophene modified carbon cloth platform. Sens. Actuat. B Chem..

[5] Nazari Z, Es’haghi Z (2022). A new electrochemical sensor for the simultaneous detection of morphine and methadone based on thioglycolic acid decorated CdSe doped graphene oxide multilayers. Anal. Bioanal. Electrochem..

[6] Mani V (2021). Electrochemical sensors targeting salivary biomarkers: A comprehensive review. TrAC Trends Anal. Chem..

[7] Yousefi N, Irandoust M, Haghighi M (2020). New and sensitive magnetic carbon paste electrode for voltammetry determination of morphine and methadone. J. Iran. Chem. Soc..

[8] Zhang C (2017). Development of quantum dots-labeled antibody fluorescence immunoassays for the detection of morphine. J. Agric. Food Chem..

[9] Caplehorn JR, Drummer OH (2002). Methadone dose and post-mortem blood concentration. Drug Alcohol Rev..

[10] Zhao P (2022). Hemin-functionalized microfluidic chip with dual-electric signal outputs for accurate determination of uric acid. ACS Appl. Mater. Interfaces.

[11] Wester N (2019). Simultaneous detection of morphine and codeine in the presence of ascorbic acid and uric acid and in human plasma at nafion single-walled carbon nanotube thin-film electrode. ACS Omega.

[12] Jalalvand AR (2020). Four-dimensional voltammetry: An efficient strategy for simultaneous determination of ascorbic acid and uric acid in the presence of dopamine as uncalibrated interference. Sens. Bio-Sens. Res..

[13] Elumalai S, Mani V, Jeromiyas N, Ponnusamy VK, Yoshimura M (2020). A composite film prepared from titanium carbide Ti _3_C_2_Tx (MXene) and gold nanoparticles for voltammetric determination of uric acid and folic acid. Microchim. Acta.

[14] Scendoni R (2022). Detection of morphine and opioids in fingernails: Immunohistochemical analysis and confirmation with ultra-high-performance liquid chromatography coupled with high-resolution mass spectrometry. Toxics.

[15] Hummel D, Löffler D, Fink G, Ternes TA (2006). Simultaneous determination of psychoactive drugs and their metabolites in aqueous matrices by liquid chromatography mass spectrometry. Environ. Sci. Technol..

[16] Li W, Li X, Yang T, Guo X, Song Y (2020). Detection of saliva morphine using surface-enhanced Raman spectroscopy combined with immunochromatographic assay. J. Raman Spectrosc..

[17] Herniman JM, Worsley PR, Greenhill R, Bader DL, John Langley G (2022). Development of ultra-high-performance supercritical fluid chromatography-mass spectrometry assays to analyze potential biomarkers in sweat. J. Sep. Sci..

[18] Pulgarín JAM, Bermejo LFG, Gallego JML, García MNS (2008). Simultaneous stopped-flow determination of morphine and naloxone by time-resolved chemiluminescence. Talanta.

[19] Zanfrognini B, Pigani L, Zanardi C (2020). Recent advances in the direct electrochemical detection of drugs of abuse. J. Solid State Electrochem..

[20] Abraham P (2020). Review on the progress in electrochemical detection of morphine based on different modified electrodes. J. Electrochem. Soc..

[21] Razlansari M (2022). Nanobiosensors for detection of opioids: A review of latest advancements. Eur. J. Pharm. Biopharm..

[22] Mohan AA, Krishna PH, Anish NR, Rasheed PA (2022). A review on advances in the developments of electrochemical sensors for the detection of anesthetic drugs. Anal. Methods.

[23] Esmaeili C (2019). A FFT square wave voltammetry sensing method for highly sensitive detection of phytic acid using a cerium oxide nanoparticles decorated graphene oxide. J. Electrochem. Soc..

[24] Asgharian Marzabad M, Jafari B, Norouzi P (2020). Determination of riboflavin by nanocomposite modified carbon paste electrode in biological fluids using fast fourier transform square wave voltammetry. Int. J. Eng..

[25] Ozer T, Henry CS (2021). Recent advances in sensor arrays for the simultaneous electrochemical detection of multiple analytes. J. Electrochem. Soc..

[26] Pakchin PS, Nakhjavani SA, Saber R, Ghanbari H, Omidi Y (2017). Recent advances in simultaneous electrochemical multi-analyte sensing platforms. TrAC Trends Anal. Chem..

[27] Benjamin SR, Junior EJMR (2022). Graphene based electrochemical sensors for detection of environmental pollutants. Curr. Opin. Environ. Sci. Health.

[28] Kim S-K, Koo H-J, Liu J, Braun PV (2017). Flexible and wearable fiber microsupercapacitors based on carbon nanotube–agarose gel composite electrodes. ACS Appl. Mater. Interfaces.

[29] Zhu J, Xiao P, Li H, Carabineiro SA (2014). Graphitic carbon nitride: Synthesis, properties, and applications in catalysis. ACS Appl. Mater. Interfaces.

[30] Asaduzzaman M (2023). A hybridized nano-porous carbon reinforced 3D graphene-based epidermal patch for precise sweat glucose and lactate analysis. Biosens. Bioelectron..

[31] Lu C, Chen X (2021). Nanostructure engineering of graphitic carbon nitride for electrochemical applications. ACS Nano.

[32] Khushaim W (2022). Porous graphitic carbon nitrides integrated biosensor for sensitive detection of cardiac troponin I. Biosens. Bioelectron. X.

[33] Mousavi M, Habibi-Yangjeh A, Pouran SR (2018). Review on magnetically separable graphitic carbon nitride-based nanocomposites as promising visible-light-driven photocatalysts. J. Mater. Sci. Mater. Electron..

[34] Farzin F, Rofouei MK, Mousavi M, Ghasemi JB (2022). A novel Z-scheme oxygen-doped g-C_3_N_4_ nanosheet/NaBiS2 nanoribbon for efficient photocatalytic H_2_O_2_ production and organic pollutants degradation. J. Phys. Chem. Solids.

[35] Sun L (2017). Cross-linked graphitic carbon nitride with photonic crystal structure for efficient visible-light-driven photocatalysis. ACS Appl. Mater. Interfaces.

[36] Habibi MM, Mousavi M, Shadman Z, Ghasemi JB (2022). Preparation of a nonenzymatic electrochemical sensor based on a gC_3_N_4_/MWO_4_ (M:Cu, Mn Co, Ni) composite for the determination of H_2_O_2_. N. J. Chem..

[37] Khamesan A (2022). Graphitic-C3N4/ZnCr-layered double hydroxide 2D/2D nanosheet heterojunction: Mesoporous photocatalyst for advanced oxidation of azo dyes with in situ produced H2O2. Adv. Powder Technol..

[38] Beitollahi H, Movahedifar F, Tajik S, Jahani S (2019). A review on the effects of introducing CNTs in the modification process of electrochemical sensors. Electroanalysis.

[39] Hu C, Hu S (2009). Carbon nanotube-based electrochemical sensors: Principles and applications in biomedical systems. J. Sens..

[40] Ghalkhani M, Shahrokhian S, Navabi M (2021). Development of an electrochemical sensor based on (rGO-CNT) nanocomposite for raloxifene analysis. Mater. Chem. Phys..

[41] Guo Z (2022). Highly accurate heart failure classification using carbon nanotube thin film biosensors and machine learning assisted data analysis. Biosens. Bioelectron. X.

[42] Wang H (2022). Three-dimensional g-C3N4/MWNTs/GO hybrid electrode as electrochemical sensor for simultaneous determination of ascorbic acid, dopamine and uric acid. Anal. Chim. Acta.

[43] Karimi-Harandi M-H, Shabani-Nooshabadi M, Darabi R (2022). Simultaneous determination of citalopram and selegiline using an efficient electrochemical sensor based on ZIF-8 decorated with RGO and g-C3N4 in real samples. Anal. Chim. Acta.

[44] Nikzad-Langerodi R, Zellinger W, Lughofer E, Saminger-Platz S (2018). Domain-invariant partial-least-squares regression. Anal. Chem..

[45] Dang VH (2020). Multivariate calibration combined differential pulse voltammetry for simultaneous electroanalytical determination of phenolic compounds using a Fe 3 O 4-modified carbon paste electrode. J. Solid State Electrochem..

[46] Moghaddam MR, Norouzi P, Ghasemi JB (2018). Simultaneous sensitive determination of benzenediol isomers using multiwall carbon nanotube–ionic liquid modified carbon paste electrode by a combination of artificial neural network and fast Fourier transform admittance voltammetry. N. J. Chem..

[47] Habibi MM, Ghasemi JB, Badiei A, Norouzi P (2022). Simultaneous electrochemical determination of morphine and methadone by using CMK-5 mesoporous carbon and multivariate calibration. Sci. Rep..

[48] Sharma P, Sarngan PP, Lakshmanan A, Sarkar D (2021). One-step synthesis of highly reactive gC 3 N 4. J. Mater. Sci. Mater. Electron..

[49] Zhang H (2015). Self-assembly of graphitic carbon nitride nanosheets–carbon nanotube composite for electrochemical simultaneous determination of catechol and hydroquinone. Electrochim. Acta.

[50] Cao Y, Alsharif S, El-Shafay A (2022). Preparation, suppressed the charge carriers recombination, and improved photocatalytic performance of g-C3N4/MoS2 pn heterojunction photocatalyst for tetracycline and dyes degradation upon visible light. Mater. Sci. Semicond. Process..

[51] Zhou X, Gao Q, Yang S, Fang Y (2020). Carbon nanotube@silicon carbide coaxial heterojunction nanotubes as metal-free photocatalysts for enhanced hydrogen evolution. Chin. J. Catal..

[52] Ma TY, Dai S, Jaroniec M, Qiao SZ (2014). Graphitic carbon nitride nanosheet–carbon nanotube three-dimensional porous composites as high-performance oxygen evolution electrocatalysts. Angew. Chem..

[53] Han X (2019). Identifying the activation of bimetallic sites in NiCo2S4@ g-C3N4-CNT hybrid electrocatalysts for synergistic oxygen reduction and evolution. Adv. Mater..

[54] Kashyap T, Biswasi S, Pal AR, Choudhury B (2019). Unraveling the catalytic and plasmonic roles of g-C3N4 supported Ag and Au nanoparticles under selective photoexcitation. ACS Sustain. Chem. Eng..

[55] Li J (2022). A non-enzymatic photoelectrochemical sensor based on g-C3N4@CNT heterojunction for sensitive detection of antioxidant gallic acid in food. Food Chem..

[56] GunaVathana SD, Thivya P, Wilson J, Peter AC (2020). Sensitive voltammetric sensor based on silver dendrites decorated polythiophene nanocomposite: Selective determination of L-Tryptophan. J. Mol. Struct..

