# Supplementary material for: Machine learning-enhanced drug testing for simultaneous morphine and methadone detection in urinary biofluids
Source: Sci Rep. 2024 Apr 6;14:8099. doi: 10.1038/s41598-024-58843-9 (PMC10998919; doi:10.1038/s41598-024-58843-9)
Supplement: Supplementary file 1 — Supplementary Information. [file 41598_2024_58843_MOESM1_ESM.docx]

**Machine Learning-Enhanced Drug Testing for Simultaneous Morphine and Methadone Detection in Urinary Biofluids**

Mohammad Mehdi Habibi^a^, Mitra Mousavi^a^, Maryam Shekofteh-Gohari^a^, Anita Parsaei-Khomami^a^, Monireh-Alsadat Hosseini^a^, Elnaz Haghani^b,c^, Razieh Salahandish^b,c*^, Jahan B. Ghasemi^a,b*^

*^a^ School of Chemistry, University College of Science, University of Tehran, P.O. Box 14155-6455, Tehran, Iran*

*^b^ Laboratory of Advanced Biotechnologies for Health Assessments (Lab-HA), Lassonde School of Engineering, York University, Toronto, ON, M3J 1P3, Canada*

*^c^ Department of Electrical Engineering and Computer Science, Biomedical Engineering Program, York University, 4700 Keele Street, Toronto, ON, M3J 1P3, Canada*

**SI-S1: Graphitic carbon nitride-carbon nanotubes (g-C_3_N_4_-CNT) preparation process and characterization**


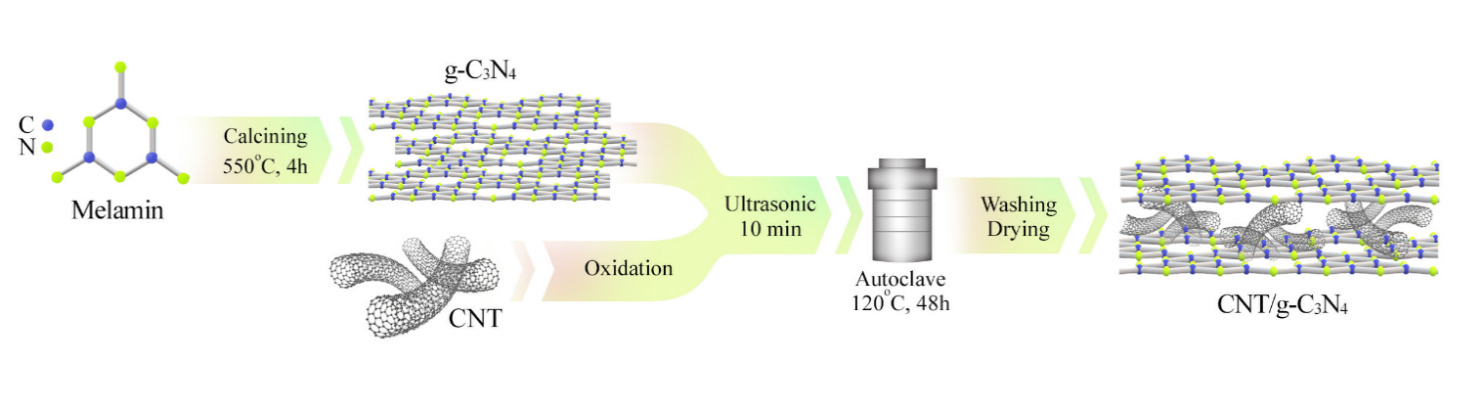


**Fig. S1.** The schematic preparation process of the graphitic carbon nitride (g-C_3_N_4_), the carboxylated process of single wall carbon nanotube (SWCNT), and the self-assembly process of g-C_3_N_4_-CNT) nanocomposite.


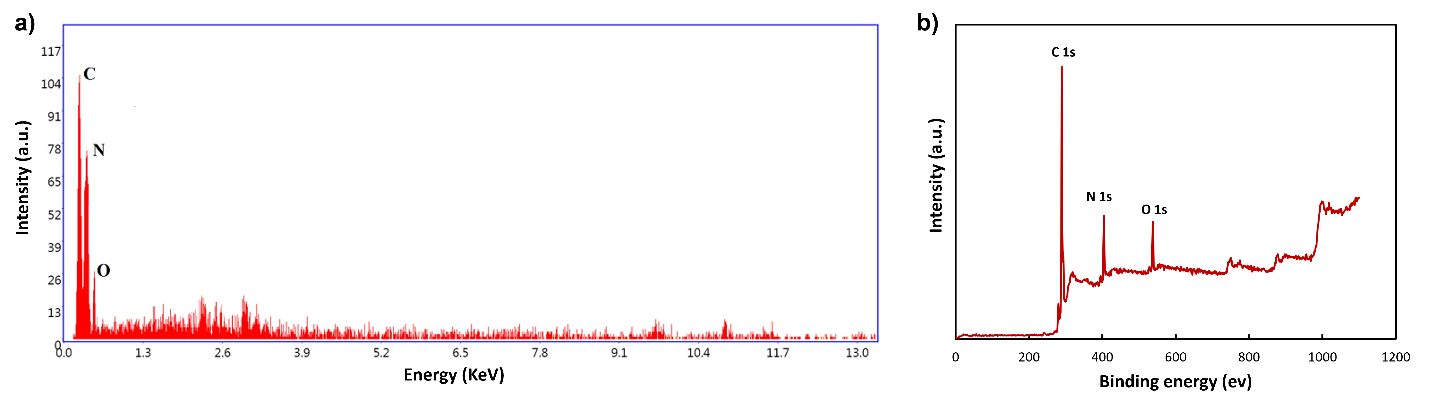


**Fig. S2.** **a)** Energy-dispersive X-ray (EDX) spectrum, **b)** the overall X-ray photoelectron spectroscopy (XPS) spectrum of g-C_3_N_4_-CNT composite.


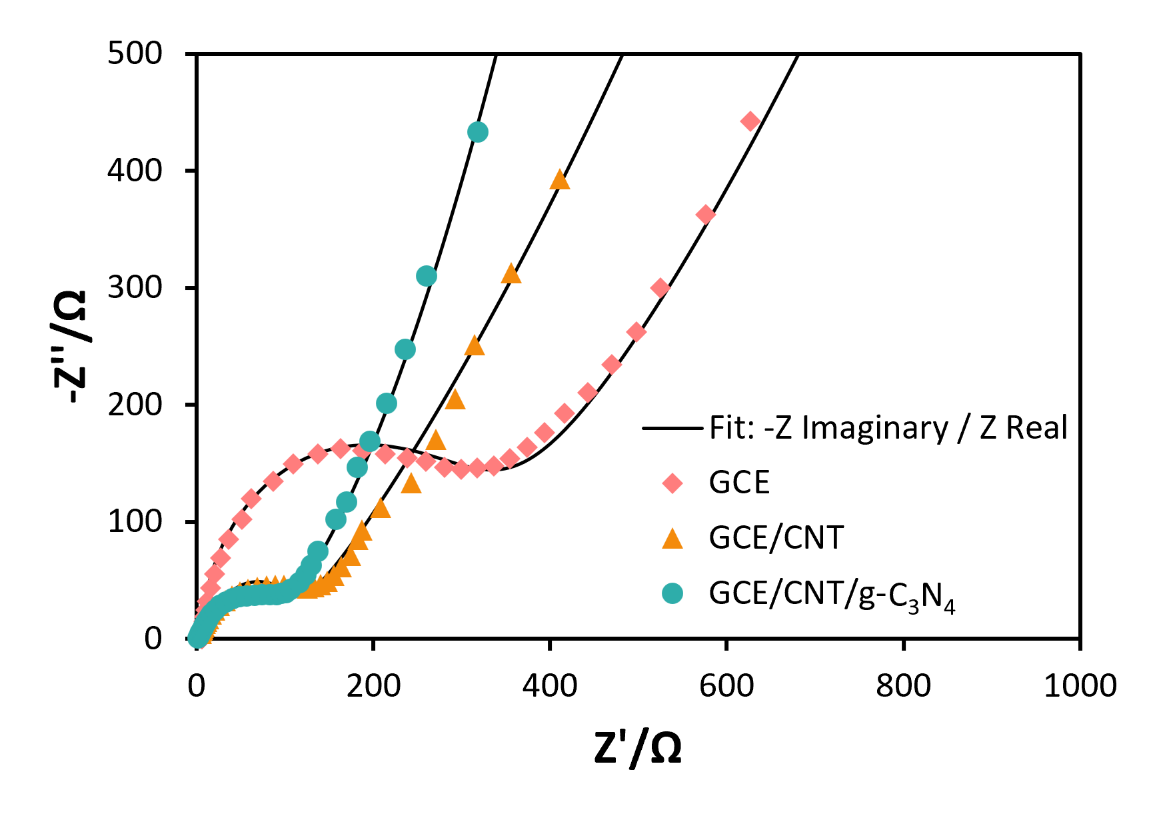


**Fig. S3.** Nyquist diagram of glassy carbon electrode (GCE), CNT-GCE, and g-C_3_N_4_-CNT-GCE at 5 mM ferrocyanide and 0.1 M potassium chloride by applying a frequency from 100 mHz to 100 kHz. The applied DC potential was 50 mV and the applied AC potential amplitude was set to 10 mV.


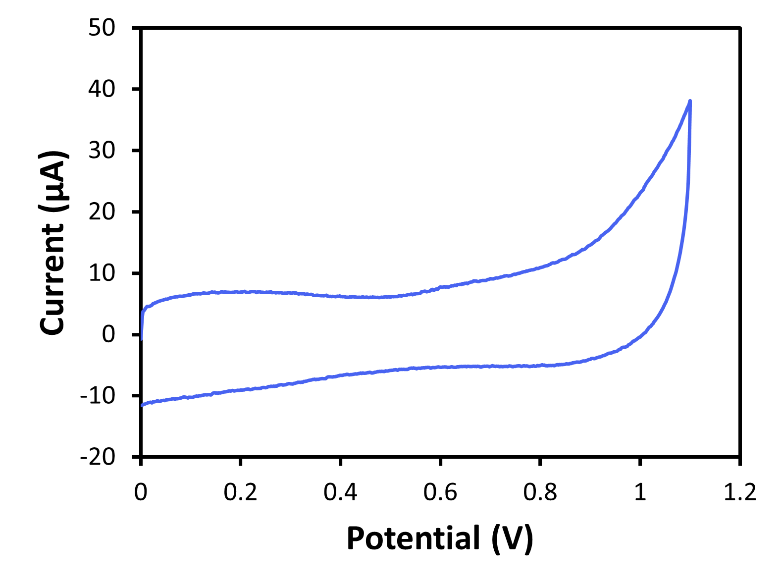


**Fig. S4.** Cyclic voltammogram of phosphate buffer solution (0.1 M and pH = 8) at the scan rate of 0.1 V/s and potential range of 0 to 1.1 V in the absence of MOR, MET, and UA.

**SI-S2: Fast Fourier transform square wave voltammetry methodology (FFT-SWV)**

FFTSWV instrument was explained in our previous works ^1-3^. This technique is an advanced electrochemical technique derived from the SWV method. A homemade potentiostat is used in connection with a personal computer to implement this technique. However, an analog-to-digital board (PCL-818H, Advantech Co.) controls the potentiostat and collects data. Also, an electrochemical software based on Delphi 6.0 performs electrochemical techniques and data processing. FFT-SWV data collection involves sampling the potential or current of the measured parameter at predefined intervals, totaling a specific number of samples. This method, a variation of SW voltammetry, applies a discrete FFT technique, background deduction, and two-dimensional integration to the electrode's response across a chosen potential range and time duration, as derived from the modified SW voltammetry. The provided figure shows the potential waveform for these measurements. This adapted approach captures current data four times during each SW polarization cycle. As indicated in the figure, the measurement waveform comprises numerous SW pulse cycles with an amplitude (E_SW_) and a frequency (fo), layered atop a stair-step potential pattern, modulated by a minor potential increment (ΔE). E_SW_ and ∆E usually fall within the range of a few mV (10 to 50 mV). The computer program calculates the count of SW cycles (N_c_) within each stair-step potential phase based on the SW frequency. The parameters of, E_SW_, Einitial, and E_final_ serve as the variables in the measurement process. It's important to note that this method captures analytical data from all electrochemical processes, encompassing charging and faradic currents. The electrode response can be computed by assessing changes in the SW voltammogram or admittance.


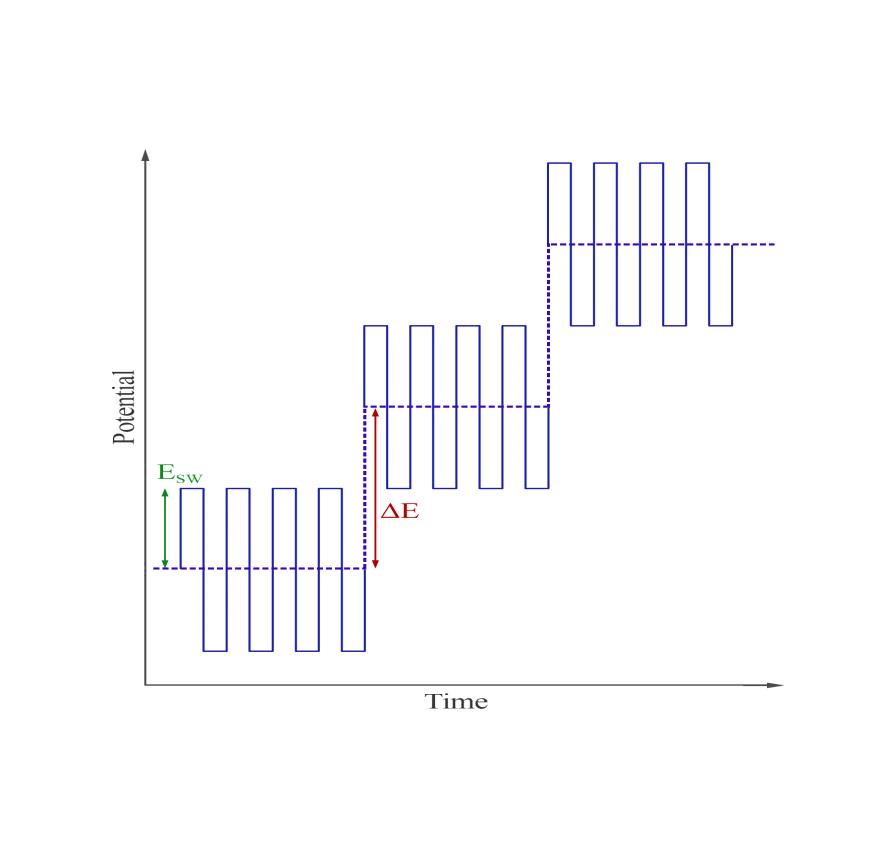


**Fig. S5.** Applied potential waveform during FFT-SWV measurements.

**SI-S3: Multivariate calibration**

The PLS method finds a set of latent variables (LVs, also called factors or components) that maximize the covariance between the X and Y spaces while minimizing the residuals (E). These LVs are linear combinations of the original variables, and the number of components is usually much smaller than the number of original variables, resulting in a simpler and more interpretable model ^4^.

Selecting the optimal number of LVs (components) in a Partial Least Squares (PLS) model is crucial for obtaining accurate predictions and avoiding overfitting. One of the common methods for selecting the optimal number of components in PLS is Cross-Validation. The dataset is divided into several subsets (often called folds), and the model is trained on a subset of the data and tested on the remaining data. This process is repeated for each fold, and performance metric (e.g., root mean square error of prediction, RMSEP) is averaged across all folds. The optimal number of components is the one that minimizes the average prediction error. The most common cross-validation techniques used in PLS are leave-one-out (LOO) and k-fold cross-validation ^1,2^.

However, in the PLS method, error calculation is crucial for assessing the model's performance and its ability to generalize to new, unseen data. Some commonly used error metrics in PLS modeling include the root mean square error (RMSE), root mean square error of cross-validation (RMSECV), and root mean square error of prediction (RMSEP). Furthermore the squared correlation coefficient (R²) is a statistical measure that represents the proportion of the variance in the dependent variable (Y) that is predictable from the independent variables (X) using the PLS model. It is used to assess the goodness of fit and the explanatory power of the PLS model.

The R^2^ value was calculated as:

$R^{2}={\sum_{i=1}^{n} {(\hat{y}-\bar{y})}^{2}}/{\sum_{i=1}^{n} {(y_{i}-\bar{y})}^{2}}$ Eq. S1

where *y*_i_ is the actual concentration of the analyte in sample *i*, $\hat{y}_{i}$ represents the estimated concentration of the analyte in sample *i*,$\bar{y}$ is the mean of the actual concentration in the calibration set, and *n* is the total number of samples used in the calibration set. The RMSEC, RMSEP and RMSECV were calculated as ^3^:

$RMSEC=\left[ \frac{1}{N}\sum_{i=1}^{n} {(y_{i}-\hat{y}_{i})}^{2} \right]^{1/2}$ Eq. S2

$RMSEP=\left[ \frac{1}{p}\sum_{i=1}^{p} {(y_{i}-\hat{y}_{i})}^{2} \right]^{1/2}$ Eq. S3

$RMSECV=\left[ \frac{1}{N}\sum_{i=1}^{n} {(y_{i}-\hat{y}_{cv})}^{2} \right]^{1/2}$ Eq. S4

**SI-S4: Simultaneous measurement of MOR**, **MET, and UA**

**Table S1.** Concentrations of MOR, MET, and UA in ternary solutions in relation to training and test samples

| **Number of samples** |  | **Concentration of samples** |  |
| --- | --- | --- | --- |
| **Training samples** | | | |
|  | **MOR (µM)** | **MET (µM)** | **UA (µM)** |
| 1 | 0 | 0 | 10 |
| 2 | 0 | 10 | 10 |
| 3 | 10 | 0 | 10 |
| 4 | 1 | 1 | 8 |
| 5 | 8 | 1 | 8 |
| 6 | 2 | 6 | 6 |
| 7 | 6 | 2 | 6 |
| 8 | 4 | 4 | 4 |
| 9 | 6 | 2 | 2 |
| 10 | 6 | 6 | 2 |
| 11 | 2 | 6 | 2 |
| 12 | 1 | 1 | 1 |
| 13 | 1 | 8 | 1 |
| 14 | 8 | 1 | 1 |
| 15 | 8 | 8 | 1 |
| 16 | 0 | 10 | 0 |
| 17 | 10 | 10 | 0 |
|  | Test samples | | |
| 18 | 2 | 1 | 0 |
| 19 | 1 | 0 | 3 |
| 20 | 4 | 6 | 0 |
| 21 | 2 | 2 | 6 |
| 22 | 1 | 0 | 8 |
| 23 | 6 | 2 | 2 |
| 24 | 10 | 10 | 0 |
| 25 | 2 | 8 | 1 |
| 26 | 3 | 1 | 4 |
| 27 | 8 | 2 | 3 |
| 28 | 2 | 0 | 10 |
| 29 | 4 | 4 | 3 |

**Table S2.**  Statistical parameters obtained of PLS for the detection of MOR, MET, and UA

| **Analytes** | **LVs** | **PLS1 RMSEC (µM)** | **RMSECV (µM)** | **PLS1 RMSEP (µM)** | **PLS1 R_p_^2^** |
| --- | --- | --- | --- | --- | --- |
| MOR | 5 | 0.1409 | 0.1827 | 0.1925 | 0.9632 |
| MET | 5 | 0.1517 | 0.1951 | 0.2035 | 0.9545 |
| UA | 5 | 0.1143 | 0.1584 | 0.1659 | 0.9651 |

A comparison of the current approach with methods described in the literature is presented in Table S4. An exhaustive literature review disclosed that no analogous records exist for the proposed technique for multivariate electrochemical simultaneous determination of MOR, MET, and UA. As illustrated in Table S4, the majority of studies were conducted independently to evaluate MOR, MET, or UA, and simultaneous measurement of these three analytes was seldom performed. Moreover, some of the previously reported techniques analyzed MOR, MET, and UA simultaneously using a univariate calibration method in which each drug was calibrated while the other operated as an interference. Because peak separation is suboptimal with these methods and the mass transfer of drugs can affect each other, this constrains the applicability of the method. However, with the multivariate calibration approach, MOR and MET can be calibrated simultaneously. Furthermore, the performance of g-C_3_N_4_-CNT combined with the multivariate calibration and FFT-SWV technique outperforms previous methods. The advantages are due to the electrocatalytic properties of g-C_3_N_4_-CNT and the ability of the PLS method to rectify the modeling and eliminate the most discordant part of the recorded electrochemical responses. In addition, the FFT-SWV technique eradicates noise (instrument noise, thermal noise, etc.) at low concentrations, improving the signal-to-noise ratio and increasing sensitivity. It is worth noting that the method used in this study is simple and provides commendable sensitivity, reproducibility, and linear range for simultaneous determination of MOR, MET, and UA.

**Table S3.** Comparison of the proposed sensor for MOR, MET, and UA with reported detection methods.

| **Ref** | **LOD (µM)** | **DLR (µM)** | **technique** | **Electrodes** | **Analytes** |
| --- | --- | --- | --- | --- | --- |
| ^5^ | 2.5 | 8-300 | DPV | Exfoliated graphene oxide/SPE | Morphine |
| ^6^ | 0.24 | 2-100 | DPV | MWCNTs/Chitosan/ GCE | Morphine, Dopamine |
| ^7^ | 0.14 | 0.34-12 | DPV | NiO/ MWCNT paste electrode | Morphine |
| ^8^ | 0.0029 | 0.008-5 | DPV | MWCNT/SnO_2_-Zn_2_SnO_4_/CPE | Morphine, Codeine |
| ^9^ | 0.4 | 0.9-400 | SWV | NiO/SWCNT/DDPM/ CPE | Morphine |
| ^10^ | 0.03 | 0.1-4 | SWV | MWCNT-Nafion/GCE | Morphine, Ondansetron |
| ^11^ | 0.009 | 0.1-310 | SWV | MWCNT/MIP/PencilGraphite Electrode | Morphine |
| ^12^ | 0.28 | 0.5-100 | DPV | MWCNT-GCE | Methadone |
| ^13^ | 0.09 | 0.1-15 | DPV | MWCNT-PGE | Methadone |
| ^14^ | 0.1 | 0.5-15 | SWV | MWCPE | Methadone |
| ^14^ | 0.005 | 0.1-500 | SWV | GNP-MWCPE | Methadone |
| ^15^ | 1 | 3-200 | DPV | nanoSnO_2_/MWCNTs/ CPE | Uric acid, Ascorbic acid, Dopamine |
| ^16^ | 0.15 | 0.5-225 | DPV | CPE/MWCNTs/IL/P dNPs | Uric acid, Ascorbic acid, Dopamine |
| ^17^ | 0.228 | 10-200 | DPV | CNNS-GO | Uric acid, Ascorbic acid, Dopamine |
| ^18^ | 0.04 | 0.5-100 | SWV | MWCNT/core-shell Fe_3_O_4_@SiO_2_ | Uric acid |
| ^19^ | 0.0083  0.0056 | 0.1-250  0.1-200 | DPV | Β-MnO_2_ nanoflowers | Morphine, Methadone |
| ^20^ | 0.0016  0.003 | 0.005-1.8  0.01-8 | SWV | graphite + MWCNT + nanomagnetic core-shell | Morphine, Methadone |
| **This work** | 0.29  0.25  0.30 | 1-15  1-15  1-20 | FFT-SWV | CNT/g-C_3_N_4_/GCE | Morphine, Methadone, Uric acid |


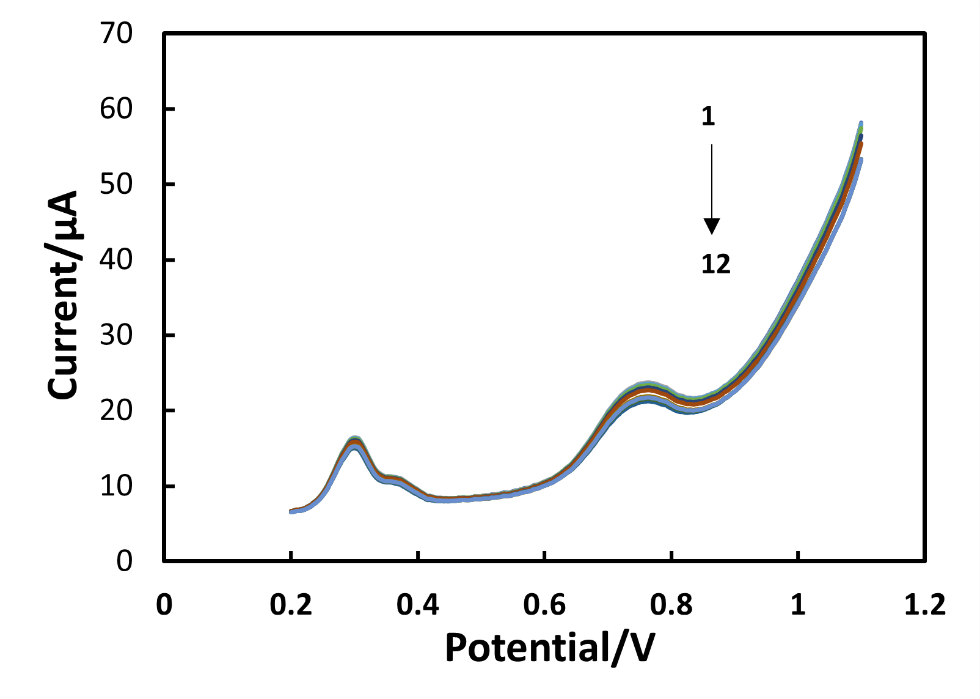


**Fig. S6.**  FFT-SWV voltammogram for 12 repeated measurements of 5 µM MOR, MET, and UA to examine the stability and reusability of the sensor.


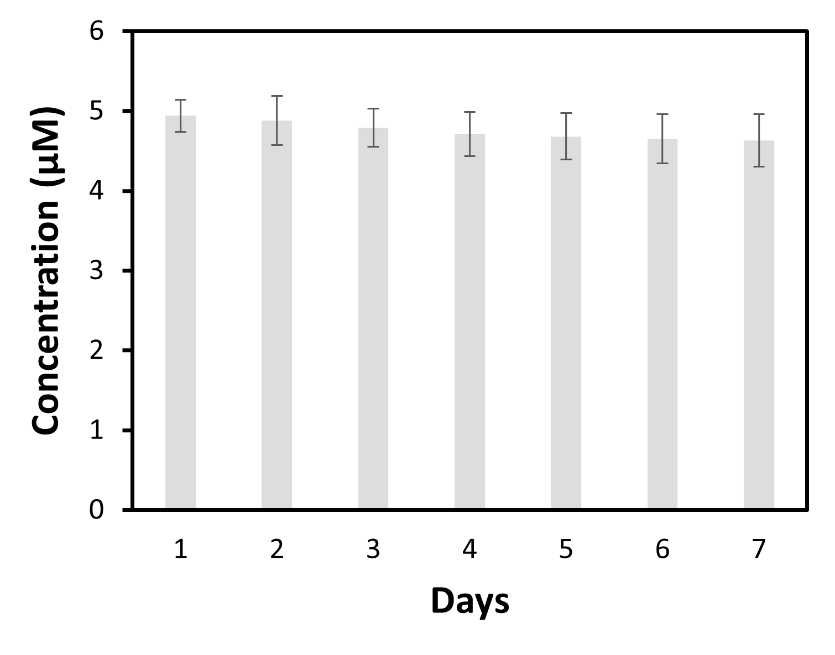


**Fig. S7.** Sensor lifetime stability test during 7 days in the presence of 5 µM MET.

**References**

1 Moghaddam, M. R., Ghasemi, J. B., Norouzi, P. & Salehnia, F. Simultaneous determination of dihydroxybenzene isomers at nitrogen-doped graphene surface using fast Fourier transform square wave voltammetry and multivariate calibration. *Microchemical Journal* **145**, 596-605 (2019).

2 Moghaddam, M. R., Norouzi, P. & Ghasemi, J. B. Simultaneous sensitive determination of benzenediol isomers using multiwall carbon nanotube–ionic liquid modified carbon paste electrode by a combination of artificial neural network and fast Fourier transform admittance voltammetry. *New Journal of Chemistry* **42**, 6479-6487 (2018).

3 Habibi, M. M., Ghasemi, J. B., Badiei, A. & Norouzi, P. Simultaneous electrochemical determination of morphine and methadone by using CMK-5 mesoporous carbon and multivariate calibration. *Scientific Reports* **12**, 8270 (2022).

4 Qin, S. J. Recursive PLS algorithms for adaptive data modeling. *Computers & Chemical Engineering* **22**, 503-514 (1998).

5 Maccaferri, G. *et al.* Highly sensitive amperometric sensor for morphine detection based on electrochemically exfoliated graphene oxide. Application in screening tests of urine samples. *Sensors and Actuators B: Chemical* **281**, 739-745 (2019).

6 Babaei, A., Babazadeh, M. & Momeni, H. A sensor for simultaneous determination of dopamine and morphine in biological samples using a multi-walled carbon nanotube/chitosan composite modified glassy carbon electrode. *International Journal of Electrochemical Science* **6**, 1382-1395 (2011).

7 Sanati, A. L., Karimi-Maleh, H., Badiei, A., Biparva, P. & Ensafi, A. A. A voltammetric sensor based on NiO/CNTs ionic liquid carbon paste electrode for determination of morphine in the presence of diclofenac. *Materials Science and Engineering: C* **35**, 379-385 (2014).

8 Taei, M., Hasanpour, F., Hajhashemi, V., Movahedi, M. & Baghlani, H. Simultaneous detection of morphine and codeine in urine samples of heroin addicts using multi-walled carbon nanotubes modified SnO2–Zn2SnO4 nanocomposites paste electrode. *Applied Surface Science* **363**, 490-498 (2016).

9 Akbarian, Y., Shabani-Nooshabadi, M. & Karimi-Maleh, H. Fabrication of a new electrocatalytic sensor for determination of diclofenac, morphine and mefenamic acid using synergic effect of NiO-SWCNT and 2, 4-dimethyl-N/-[1-(2, 3-dihydroxy phenyl) methylidene] aniline. *Sensors and Actuators B: Chemical* **273**, 228-233 (2018).

10 Nigović, B., Sadiković, M. & Sertić, M. Multi-walled carbon nanotubes/Nafion composite film modified electrode as a sensor for simultaneous determination of ondansetron and morphine. *Talanta* **122**, 187-194 (2014).

11 Rezaei, B., Foroughi-Dehnavi, S. & Ensafi, A. A. Fabrication of electrochemical sensor based on molecularly imprinted polymer and nanoparticles for determination trace amounts of morphine. *Ionics* **21**, 2969-2980 (2015).

12 Amiri-Aref, M., Raoof, J. B. & Ojani, R. Electrocatalytic oxidation and selective determination of an opioid analgesic methadone in the presence of acetaminophen at a glassy carbon electrode modified with functionalized multi-walled carbon nanotubes: application for human urine, saliva and pharmaceutical samples analysis. *Colloids and Surfaces B: Biointerfaces* **109**, 287-293 (2013).

13 Alipour, E., Majidi, M. R. & Hoseindokht, O. Development of simple electrochemical sensor for selective determination of methadone in biological samples using multi‐walled carbon nanotubes modified pencil graphite electrode. *Journal of the Chinese Chemical Society* **62**, 461-468 (2015).

14 Afkhami, A., Soltani-Felehgari, F. & Madrakian, T. A sensitive electrochemical sensor for rapid determination of methadone in biological fluids using carbon paste electrode modified with gold nanofilm. *Talanta* **128**, 203-210 (2014).

15 Sun, D., Zhao, Q., Tan, F., Wang, X. & Gao, J. Simultaneous detection of dopamine, uric acid, and ascorbic acid using SnO 2 nanoparticles/multi-walled carbon nanotubes/carbon paste electrode. *Analytical Methods* **4**, 3283-3289 (2012).

16 Rafati, A. A., Afraz, A., Hajian, A. & Assari, P. Simultaneous determination of ascorbic acid, dopamine, and uric acid using a carbon paste electrode modified with multiwalled carbon nanotubes, ionic liquid, and palladium nanoparticles. *Microchimica Acta* **181**, 1999-2008 (2014).

17 Zhang, H. *et al.* Graphitic carbon nitride nanosheets doped graphene oxide for electrochemical simultaneous determination of ascorbic acid, dopamine and uric acid. *Electrochimica Acta* **142**, 125-131 (2014).

18 Arvand, M. & Hassannezhad, M. Square wave voltammetric determination of uric acid and diclofenac on multi-walled carbon nanotubes decorated with magnetic core-shell Fe 3 O 4@ SiO 2 nanoparticles as an enhanced sensing interface. *Ionics* **21**, 3245-3256 (2015).

19 Akbari, S., Jahani, S., Foroughi, M. M. & Nadiki, H. H. Simultaneous determination of methadone and morphine at a modified electrode with 3D β-MnO 2 nanoflowers: application for pharmaceutical sample analysis. *RSC advances* **10**, 38532-38545 (2020).

20 Yousefi, N., Irandoust, M. & Haghighi, M. New and sensitive magnetic carbon paste electrode for voltammetry determination of morphine and methadone. *Journal of the Iranian Chemical Society* **17**, 2909-2922 (2020).
